# Plasma phospholipid n-3 and n-6 polyunsaturated fatty acids in relation to cardiometabolic markers and gestational diabetes: A longitudinal study within the prospective NICHD Fetal Growth Studies

**DOI:** 10.1371/journal.pmed.1002910

**Published:** 2019-09-13

**Authors:** Yeyi Zhu, Mengying Li, Mohammad L. Rahman, Stefanie N. Hinkle, Jing Wu, Natalie L. Weir, Yuan Lin, Huixia Yang, Michael Y. Tsai, Assiamira Ferrara, Cuilin Zhang

**Affiliations:** 1 Division of Research, Kaiser Permanente Northern California, Oakland, California, United States of America; 2 Department of Epidemiology & Biostatistics, University of California, San Francisco, California, United States of America; 3 Division of Intramural Population Health Research, Eunice Kennedy Shriver National Institute of Child Health and Human Development, Bethesda, Maryland, United States of America; 4 Department of Population Medicine and Harvard Pilgrim Health Care Institute, Harvard Medical School, Boston, Massachusetts, United States of America; 5 Glotech Inc., Bethesda, Maryland, United States of America; 6 Department of Laboratory Medicine and Pathology, University of Minnesota, Minneapolis, Minnesota, United States of America; 7 Department of Epidemiology, Richard M. Fairbanks School of Public Health, Indiana University, Indianapolis, Indiana, United States of America; 8 Department of Obstetrics and Gynecology, Peking University First Hospital, Beijing, China; University of Cambridge, UNITED KINGDOM

## Abstract

**Background:**

Despite dietary recommendations of polyunsaturated fatty acids (PUFAs) for cardiometabolic health, n-3 and n-6 PUFAs and their interplay in relation to diabetes risk remain debated. Importantly, data among pregnant women are scarce. We investigated individual plasma phospholipid n-3 and n-6 PUFAs in early to midpregnancy in relation to subsequent risk of gestational diabetes mellitus (GDM).

**Methods and findings:**

Within the National Institute of Child Health and Human Development (NICHD) Fetal Growth Studies–Singleton Cohort (*n* = 2,802), individual plasma phospholipid n-3 and n-6 PUFAs levels were measured at gestational weeks (GWs) 10–14, 15–26, 23–31, and 33–39 among 107 GDM cases (ascertained on average at GW 27) and 214 non-GDM controls. Conditional logistic regression was used, adjusting for major risk factors for GDM. After adjusting for covariates, individual n-3 eicosapentaenoic acid (EPA), docosapentaenoic acid (DPA), and docosahexaenoic acid (DHA) were inversely correlated with insulin-resistance markers, whereas individual n-6 dihomo-gamma-linolenic acid (DGLA) was positively correlated with insulin-resistance markers. At GW 15–26, a standard deviation (SD) increase in total n-3 PUFAs and individual n-3 DPA was associated with a 36% (adjusted odds ratio 0.64; 95% CI 0.42–0.96; *P* = 0.042) and 33% (0.67; 95% CI 0.45–0.99; *P* = 0.047) lower risk of GDM, respectively; however, the significance did not persist after post hoc false-discovery rate (FDR) correction (FDR-corrected *P* values > 0.05). Associations between total n-6 PUFAs and GDM were null, whereas associations with individual n-6 PUFAs were differential. Per SD increase, gamma-linolenic acid (GLA) at GWs 10–14 and DGLA at GWs 10–14 and 15–26 were significantly associated with a 1.40- to 1.95-fold higher risk of GDM, whereas docosatetraenoic acid (DTA) at GW 15–26 was associated with a 45% (0.55; 95% CI 0.37–0.83) lower risk of GDM (all FDR-corrected *P* values < 0.05). Null associations were observed for linoleic acid (LA) in either gestational window in relation to risk of GDM. Women with high (≥median) n-3 PUFAs and low (<median) n-6 PUFAs levels had a 64% (95% CI 0.14–0.95; *P* value = 0.039) lower risk of GDM versus women with low n-3 and high n-6 PUFAs. Limitations include the inability to distinguish between exogenous and endogenous influences on circulating PUFA levels and the lack of causality inherent in observational studies.

**Conclusions:**

Our findings may suggest a potential role of primarily endogenously metabolized plasma phospholipid n-6 PUFAs including GLA, DGLA, and DTA in early to midpregnancy in the development of GDM. Null findings on primarily diet-derived n-3 EPA and DHA and n-6 LA do not provide strong evidence to suggest a beneficial role in prevention of GDM, although not excluding the potential benefit of EPA and DHA on glucose–insulin homeostasis given the inverse associations with insulin-resistance markers. Our findings highlight the importance of assessing individual circulating PUFAs to investigate their distinct pathophysiologic roles in glucose homeostasis in pregnancy.

## Abstract 2

Cuilin Zhang and colleagues reveal a potential association between polyunsaturated fatty acids in early to midpregnancy with the development of gestational diabetes.

## Author summary

**Why was this study done?:**

- Dietary guidelines promote intakes of polyunsaturated fatty acids (PUFAs) for cardiometabolic health, whereas evidence on PUFAs for pregnant women is scarce.
- Experimental and observational studies have linked both dietary and circulating fatty acids to the risk of developing type 2 diabetes, insulin resistance, and β-cell dysfunction, but only a few studies did for the risk of developing gestational diabetes mellitus (GDM).
- In particular, evidence is missing for the associations of individual plasma phospholipid PUFAs, rather than total PUFAs conventionally via dietary assessment, with the risk of subsequent GDM.

**What did the researchers do and find?:**

- In this longitudinal study of 107 women with GDM and 214 matched non-GDM women within the prospective National Institute of Child Health and Human Development (NICHD) Fetal Growth Studies–Singleton Cohort in the United States, we evaluated the associations of individual plasma phospholipid PUFAs in early to midpregnancy with a panel of glucose metabolism and cardiometabolic markers and subsequent risk of GDM.
- Total and individual plasma phospholipid n-3 PUFAs—specifically, diet-derived eicosapentaenoic acid and docosahexaenoic acid—were inversely correlated with insulin-resistance markers but not associated with the risk of GDM.
- Associations of individual plasma phospholipid n-6 PUFAs with GDM risk were differential, with no associations for the major diet-derived n-6 PUFAs (linolenic acid and arachidonic acid) comprising over 80% of total n-6 PUFAs and with null, positive, or negative associations for minor n-6 PUFAs recognized to be primarily produced from endogenous metabolism of linolenic acid.

**What do these findings mean?:**

- No significant associations of major diet-derived n-6 PUFAs and significant findings for the other minor n-6 PUFAs known to reflect endogenous metabolism suggest a potential role of circulating levels of plasma phospholipid n-6 PUFAs in pathophysiology of GDM.
- Furthermore, our findings do not provide strong evidence to suggest beneficial roles of primarily diet-derived n-3 PUFAs in prevention of GDM, although also not excluding the possibility of benefit on glucose–insulin homeostasis given their inverse correlations with insulin-resistance markers. Similarly, our findings suggest neither a harmful nor a beneficial role of diet-derived n-6 PUFA linolenic acid in GDM pathophysiology.
- Our findings among pregnant women highlight the need to recognize the heterogeneous associations of individual circulating PUFAs with subsequent GDM risk and to investigate the underlying metabolism.

## Introduction

Gestational diabetes mellitus (GDM) has emerged as the most common metabolic complication, affecting 7%–25% of pregnancies worldwide [1,2], forming a growing, urgent public health concern [3]. Nutritional perturbations have been implicated in the programming of glucose homeostasis [4], with carbohydrate metabolism conceivably serving as a key component given its impact on postprandial glucose and insulin responses [5]. Notably, emerging experimental data have linked altered fatty acids composition to exacerbation of insulin resistance and β-cell dysfunction [6–8], suggesting additional pathways underlying the etiology of hyperglycemia.

Indeed, recognizing the importance of dietary fatty acids composition, dietary guidelines promote intakes of polyunsaturated fatty acids (PUFAs) for cardiometabolic health [9,10], whereas epidemiological data on glucose homeostasis and diabetes risk remain equivocal. Recent meta-analyses suggest heterogeneous findings. Randomized clinical trials suggest a lack of effect of dietary n-3 PUFAs on markers of glucose–insulin homeostasis [11,12]. In observational studies, dietary or biomarker n-3 PUFAs overall do not appear to be associated with type 2 diabetes mellitus (T2DM) risk, although protective associations have been observed in Asian populations [13–16]. On the other hand, dietary n-6 PUFAs exhibited to improve glucose–insulin homeostasis in two recent meta-analyses of randomized controlled feeding studies [17,18], consistent with the suggestive evidence for an inverse association between biomarker linoleic acid (LA), the most abundant form of PUFAs, and T2DM [19]. Nonetheless, the link between dietary n-6 PUFAs and cardiometabolic outcomes in observational studies and trials remains elusive [20–22]. Yet, beneath all these data were important limitations that undermined the evidence: inevitable measurement errors due to dietary assessment via subjective report, differences in exposure assessment window, predominantly white study populations, and inability to disentangle potentially distinct health effects of individual PUFAs. Indeed, except for the essential fatty acids alpha-linolenic acid (ALA) and LA, levels of circulating long-chain PUFA derivatives are functions of both exogenous (via dietary intake) and endogenous (via lipogenesis) origins, with unique biochemical properties and metabolic effects [23]. Further, data among pregnant women remain scarce, with only one prospective examination of serum PUFA in second trimester with GDM risk in a small sample including 49 GDM women [24]. These limitations call for longitudinal objective measurement of individual and subclasses of circulating PUFAs among multiracial/ethnic populations to advance our understanding of their potentially distinct pathophysiologic roles in the development of GDM. Moreover, despite the dynamic physiologic alterations during pregnancy, variation of the phospholipid PUFAs profile throughout pregnancy is as-yet understudied.

To address these critical knowledge gaps, we aimed to prospectively investigate the associations of individual circulating PUFAs in early to midpregnancy with a comprehensive panel of glucose metabolism and cardiometabolic markers and subsequent risk of GDM. We hypothesized that higher levels of individual plasma phospholipid n-3 PUFAs in early to midpregnancy are associated with lower risk of GDM and optimal glucose homeostasis and cardiometabolic biomarker profile, that an opposite pattern may exhibit for individual n-6 PUFAs, and that these associations may vary by gestational window of exposure because of potential changes in physiology and maternal–fetal transfer of plasma phospholipid PUFAs as pregnancy progressed [25]. We also aimed to characterize the longitudinal profiles of plasma phospholipid PUFAs across gestation.

## Methods

### Study population and design

The participants were from the Eunice Kennedy Shriver National Institute of Child Health and Human Development (NICHD) Fetal Growth Studies–Singleton Cohort, a prospective multiracial study of low-risk, singleton pregnant women [26]. In brief, 2,802 pregnant women (2,334 nonobese and 468 obese) aged 18–40 years with prepregnancy body mass index (BMI) ranging from 19.0 to 44.9 kg/m^2^ and free of preexisting diseases (i.e., hypertension, diabetes, renal/autoimmune disease, psychiatric disorder, cancer, or HIV/AIDs) were recruited between gestational weeks 8 and 13 at 12 clinical centers across the US (2009–2013). The study was approved by the institutional review boards of all participating institutions. Written informed consent was obtained from all participants.

In the entire prospective cohort, we identified 107 GDM cases via medical record review according to the Carpenter and Coustan criteria based on the definition recommended by American Diabetes Association and American College of Obstetricians and Gynecologists [27]. The average (± SD) gestational age at the 100-g, 3-hour oral glucose tolerance test for GDM diagnosis among cases was 27 (± 4) weeks. Women with GDM were individually matched at a ratio of 1:2 to 214 non-GDM controls as part of the GDM etiology study within the NICHD Fetal Growth Studies (S1 Fig; S1 Protocol) [28]. Matching variables included age (± 2 years), race/ethnicity (non-Hispanic white, non-Hispanic black, Hispanic, Asian/Pacific Islander), and gestational week of blood sample collection (± 2 weeks). Maternal blood specimens were longitudinally collected four times during pregnancy targeted at gestational weeks 8–13, 16–22, 24–29, and 34–37 (actual range: 10–14, 15–26, 23–31, and 33–39 weeks, without within-participant overlapping). At 15–26 weeks, samples were collected after an overnight fast of 8–14 hours among both cases and controls. The fasting duration prior to biospecimen collection at all visits was similar between cases and controls. Biospecimens were stored at −80 °C until being thawed immediately before assay. By design, participants were randomized to different gestational weeks within each time window of blood collection. This mixed longitudinal randomization scheme maximized the opportunity of capturing weekly data without exposing women to weekly examinations and allowed evaluation of gestational patterns of plasma phospholipid PUFAs within smaller gestational-age intervals of 3–4 weeks, as done previously [26,29,30].

### Biomarker assessment

We collected a total of four blood samples per woman in the entire cohort, specifically two before, one around, and one after the diagnosis of GDM, with at least one collection per trimester to allow the assessment of clinically relevant trimester-specific physiologic alterations across pregnancy. From the first two blood collections (i.e., weeks 10–14 [median 13, interquartile range (IQR) 12–14] and 15–26 [median 19, IQR 18–21]), biomarkers were measured among all the cases (*n* = 107) and controls (*n* = 214). To ensure that biomarker measurements at these two visits preceded the diagnosis of GDM, we excluded one case at weeks 10–14 and five cases at 15–26 weeks from the final analysis, whose blood samples were collected after the diagnosis of GDM. From blood collections at the two subsequent visits (i.e., weeks 23–31 [median 27, IQR 25–28] and 33–39 [median 36, IQR 35–37]), biomarkers were measured among cases and one of their randomly (consistent at both visits) selected controls (both *n* = 107). The average (± SD) within-participant, nonoverlapping gaps between the four visits were 6.5 (± 2.3), 7.6 (± 1.5), and 9.0 (± 1.5) weeks, respectively.

Plasma phospholipid PUFAs were measured using a Hewlett Packard 5890 gas chromatography system as described previously [31]. Briefly, total lipids were extracted from plasma, and phospholipid fraction was separated using thin-layer chromatography. Isolated phospholipids were then converted to fatty acid methyl esters for further separation by gas chromatograph. Fatty acids were identified using mixtures of known fatty acid methyl esters purchased from Nu-Chek Prep (Elysian Township, MN, USA). Among 28 fatty acids with relative percentage above 0.05%, which collectively covered up to 98% of the peaks measured, we identified 11 PUFAs, including four n-3 PUFAs: 18:3n-3 (ALA), 20:5n-3 (eicosapentaenoic acid [EPA]), 22:5n-3 (n-3 docosapentaenoic acid [DPA]), and 22:6n-3 (docosahexaenoic acid [DHA]); and seven n-6 PUFAs: 18:2n-6 (LA), 18:3n-6 (gamma-linolenic acid [GLA]), 20:2n-6 (eicosadienoic acid [EDA]), 20:3n-6 (dihomo-gamma-linolenic acid [DGLA]), 20:4n-6 (arachidonic acid [AA]), 22:4n-6 (docosatetraenoic acid [DTA]), and 22:5n-6 (n-6 DPA). The content of individual plasma phospholipid PUFA was expressed as a percentage (%) of the total phospholipid fatty acids. Samples of matched case–control pairs were assayed in the same analytic run in a random order. Interassay coefficients of variation (CVs) were assessed using in-house pooled control samples obtained from pregnant women and measured with each batch, totaling 40 repeats. The CVs for individual plasma phospholipid PUFAs reported in this study were <11% for all (ALA 10.9%, n-3 DPA 10.4%, DHA 5.4%, LA 2.8%, GLA 10.7%, EDA 5.9%, DGLA 4.5%, AA 4.8%, n-6 DPA 9.6%) but EPA (22.1%) and DTA (29.9%), which had relatively low abundance in pregnancy. The reported interassay CVs were consistent with previously reported ones among pregnant [32,33] and nonpregnant individuals [34]. Individual PUFAs were also grouped into subclasses of n-3 or n-6 PUFAs (see biosynthesis pathways and sources of individual PUFAs in S2 Fig). We also derived ratios of product to precursor PUFAs as indicators of fatty acid elongase and desaturase enzyme activities, which have been implicated in lipid metabolism and insulin action [35]: 18:3n-6/18:2n-6 (GLA/LA) indicating Δ6-desaturase activity catalyzing the conversion of LA to GLA, 20:4n-6/20:3n-6 (AA/DGLA) indicating Δ5-desaturase activity catalyzing the conversion of DGLA to AA, and 20:3n-6/18:2n-6 (DGLA/LA) indicating the conversion of LA to DGLA.

Further, we measured a panel of glucose metabolism and cardiometabolic biomarkers including glucose, insulin, C-peptide, high-sensitivity C-reactive protein (hs-CRP), high-molecular-weight (HMW) adiponectin, leptin, total cholesterol, low-density lipoprotein cholesterol (LDL-C), high-density lipoprotein cholesterol (HDL-C), and triglycerides. Specifically, concentrations of plasma glucose and hs-CRP were measured by enzymatic assays using the Roche Modular P Chemistry analyzer; total cholesterol, HDL-C, and triglycerides by Roche COBAS 6000 Chemistry Analyzer; and insulin and C-peptide by Roche Elecsys 2010 Analyzer (Roche Diagnostics, Indianapolis, IN, USA). Concentrations of LDL-C were calculated by the Friedewald’s formula: LDL-C = total cholesterol − HDL-C—triglycerides / 5 [36]. All values of plasma lipid levels were expressed in mg/dl. We also measured plasma HMW adiponectin using a quantitative sandwich enzyme immunoassay (Beckman Coulter, Fullerton, CA, USA) and leptin by the Mercodia Leptin ELISA (Mercodia AB, Uppsala, Sweden). The interassay CVs of these biomarkers were all less than 6.0%. All assays were performed without knowledge of the case–control status.

### Covariates

Data on maternal demographic, lifestyle, and clinical factors were obtained from structured questionnaires and medical records. Covariates were a priori selected including conventional risk factors for GDM: parity (nulliparous, multiparous), family history of diabetes (yes, no), and prepregnancy BMI (<25.0, 25.0–29.9, 30.0–34.9, 35.0–44.9 kg/m^2^). In this low-risk population, nonobese women who smoked in the 6 months preceding the index pregnancy were ineligible, and only five obese women reported smoking in the 6 months before pregnancy. Thus, smoking was not included as a covariate. Given that maternal age (years) and gestational week of blood collection (weeks) were matched between cases and controls within a certain range, we also included these two matching factors as continuous variables to derive conservative risk estimates.

### Statistical methods

Differences between cases and controls in participant characteristics and plasma phospholipid PUFA concentrations (%) and fatty acid ratios at the two visits prior to GDM diagnosis were assessed by linear mixed models, with associated likelihood ratio tests for continuous variables, and by logistic regression with generalized estimating equations for categorical variables, including participant-specific random intercepts and a random effect term for matched case–control pairs. Longitudinal trends of PUFAs and fatty acid ratios throughout pregnancy were plotted according to gestational-age intervals among women with and without GDM, with between-group comparisons obtained by linear mixed models with aforementioned model specifications.

To gain mechanistic insight into the potential underlying pathophysiological processes involved in glucose homeostasis, we calculated partial Spearman’s correlation coefficients of individual and subclasses of circulating PUFAs and fatty acid ratios at gestational weeks 10–14 with markers of glucose homeostasis (fasting plasma glucose, insulin, C-peptide, hs-CRP, and homeostasis model assessment of insulin resistance [HOMA-IR] [37]) and cardiometabolic risk (adiponectin, leptin, total cholesterol, HDL-C, LDL-C, and triglycerides) at the subsequent visit (i.e., gestational weeks 15–26), adjusting for covariates. Heat maps were also created to visualize and evaluate the cross-sectional correlations among individual PUFAs and fatty acid ratios and aforementioned biomarkers at weeks 10–14 among non-GDM controls.

Multivariable conditional logistic regression models were fitted to assess the associations of individual circulating PUFAs and fatty acid ratios at the first two visits (gestational weeks 10–14 and 15–26) with risk of GDM, respectively, adjusting for aforementioned covariates. We analyzed each PUFA and fatty acid ratio as a categorical variable in quartiles based on the distribution among controls to allow examination of potentially nonlinear associations, and also a continuous variable per standard deviation (SD). Tests of linear trend were conducted by using the median value for each quartile and fitted as a continuous variable in the conditional logistic regression models. Given the overall consistent findings in the quartile-specific and continuous exposure models, we presented the latter as primary results. Similar analyses were conducted for PUFA subclasses (i.e., n-3 and n-6 PUFAs). Further, to investigate the potential interplay of PUFA subclasses in relation to GDM risk, we assessed the risk estimates associated with joint categories of high or low n-3 and n-6 PUFAs determined as above or below the respective median at the first two visits, including an interaction term for continuous n-3 and n-6 PUFAs examined by the likelihood ratio test. We also assessed the association of longitudinal trends of plasma phospholipid PUFAs and ratios from weeks 10–14 to 15–26 with GDM risk by fitting generalized linear mixed models with participant-specific random intercepts, an autoregressive covariance structure, and a random effect for the matched case–control pairs. Post hoc multiple-comparison adjustment for *P* values was performed using the Benjamini-Hochberg false-discovery rate (FDR)-controlling method [38]. In sensitivity analysis, we additionally adjusted for plasma phospholipid saturated fatty acids (SFAs) to explore the potential impact of interplay with SFAs on the PUFA-GDM association. We also excluded three women with undiagnosed preexisting diabetes using hemoglobin A1C (≥6.5%) at weeks 10–14. In addition, we included cross-product terms to evaluate whether the associations of circulating PUFAs and fatty acid ratios with subsequent GDM risk were modified by major risk factors for GDM (prepregnancy obesity status, family history of diabetes, and race/ethnicity). All analyses were conducted using SAS version 9.4 (SAS Institute, Cary, NC, USA), and significance defined as a two-tailed *P* value < 0.05.

## Results

Compared to non-GDM controls, women with GDM were more likely to have a family history of diabetes and be overweight or obese before pregnancy (Table 1). Among all PUFAs, LA (18:2n-6) was the most abundant form and accounted for 21%–22% of the total plasma phospholipid fatty acids level, followed by AA 20:4n-6 for 10%–11%, DHA for 4%, and DGLA 20:3n-6 for 3%–4%, whereas other individual PUFAs contributed less than 1%, with the lowest concentration observed for GLA (0.07%–0.08%) at gestational weeks 10–14 and 15–26 (Table 2). Compared to non-GDM controls, women with GDM had significantly lower levels of DHA at weeks 10–14, EPA at weeks 15–26, total n-3 PUFAs at both weeks 10–14 and 15–26, and EDA and DTA at weeks 15–26, whereas they had significantly higher levels of GLA at weeks 10–14 and DGLA at weeks 10–14 and 15–26. Among PUFA ratios, Δ6-desaturase at weeks 10–14 and DGLA/LA at both gestational periods were significantly higher, whereas Δ5-desaturase was significantly lower among GDM women compared to non-GDM controls.

**Table 1:** Participant characteristics among women with GDM and their matched controls, the NICHD Fetal Growth Studies–Singleton Cohort^a^.

	GDM cases (*n* = 107)	Non-GDM controls (*n* = 214)	*P* value^b^
Age (years)	30.5 ± 5.7	30.4 ± 5.4	
Race/ethnicity			
Non-Hispanic white	25 (23.4)	50 (23.4)	
Non-Hispanic black	15 (14.0)	30 (14.0)	
Hispanic	41 (38.3)	82 (38.3)	
Asian/Pacific Islander	26 (24.3)	52 (24.3)	
Education			0.18
Less than high school	17 (15.9)	26 (12.1)	
High school graduate or equivalent	15 (14.0)	23 (10.7)	
More than high school	75 (70.1)	165 (77.1)	
Insurance			0.43
Private or managed care	68 (63.5)	143 (66.8)	
Medicaid, self-pay, or other	39 (36.5)	71 (33.1)	
Marital status			0.12
Never married	11 (10.3)	35 (16.4)	
Married/living with a partner	92 (86.0)	167 (78.0)	
Divorced/separated	4 (3.7)	12 (5.6)	
Nulliparity	48 (44.9)	96 (44.9)	1
Family history of diabetes	40 (37.4)	48 (22.4)	0.003
Prepregnancy body mass index, kg/m^2^			<0.001
Normal weight, 19.0–24.9	37 (34.6)	123 (58.0)	
Overweight, 25.0–29.9	35 (32.7)	56 (26.4)	
Obese class 1, 30.0–34.9	20 (18.7)	17 (8.0)	
Obese classes 2 and 3, 35.0–44.9	15 (14.0)	16 (7.6)	
Smoking 6 months preconception	4 (3.7)	1 (0.5)	0.06
Alcoholic beverage consumption 3 months preconception	61 (57.0)	137 (64.0)	0.22

^a^Data are presented as *n* (%) for categorical variables and mean (SD) for continuous variables, unless otherwise specified.

^b^Obtained by linear mixed models with associated likelihood ratio tests for continuous variables and binomial/multinomial logistic regression with generalized estimating equations for binary/multilevel categorical variables (Wald tests), accounting for matched case–control pairs. *P* values are not shown for matching variables, age, and race/ethnicity.

Abbreviations: GDM, gestational diabetes mellitus; NICHD, National Institute of Child Health and Human Development; SD, standard deviation

**Table 2:** Distribution of plasma phospholipid n-3 PUFA (%), n-6 PUFA (%), and PUFA ratios among GDM cases and non-GDM controls^a^.

	Gestational weeks 10–14			Gestational weeks 15–26		
	Controls (*n* = 214)	GDM (*n* = 107)	*P*^b^	Controls (*n* = 214)	GDM (*n* = 107)	*P*^b^
**n-3 PUFAs, % of total fatty acids**						
18:3n-3 (ALA)	0.20 (0.16, 0.26)	0.21 (0.17, 0.26)	0.62	0.24 (0.20, 0.29)	0.25 (0.19, 0.28)	0.73
20:5n-3 (EPA)	0.29 (0.21, 0.4)	0.29 (0.19, 0.43)	0.74	0.16 (0.14, 0.21)	0.15 (0.12, 0.19)	0.047
22:5n-3 (DPA)	0.68 (0.55, 0.81)	0.65 (0.54, 0.80)	0.25	0.62 (0.51, 0.76)	0.58 (0.46, 0.7)	0.031
22:6n-3 (DHA)	4.16 (3.37, 5.13)	3.92 (3.05, 4.73)	0.049	3.99 (3.23, 5.00)	3.71 (2.97, 4.91)	0.085
Total n-3 PUFAs	5.40 (4.58, 6.41)	5.11 (4.14, 6.12)	0.047	5.10 (4.17, 6.21)	4.70 (3.87, 5.99)	0.047
**n-6 PUFAs, % of total fatty acids**						
18:2n-6 (LA)	20.59 (18.85, 22.54)	20.50 (18.74, 22.06)	0.510	21.59 (19.91, 23.33)	21.57 (19.78, 23.29)	0.990
18:3n-6 (GLA)	0.07 (0.06, 0.09)	0.08 (0.06, 0.11)	0.010	0.07 (0.05, 0.09)	0.07 (0.06, 0.09)	0.890
20:2n-6 (EDA)	0.50 (0.44, 0.57)	0.47 (0.43, 0.56)	0.110	0.52 (0.45, 0.59)	0.49 (0.43, 0.58)	0.027
20:3n-6 (DGLA)	3.41 (2.83, 4.01)	3.66 (3.20, 4.53)	<0.0013	3.35 (2.88, 3.91)	3.75 (3.30, 4.32)	<0.0011
20:4n-6 (AA)	11.34 (9.91, 12.54)	10.88 (9.42, 12.63)	0.330	10.42 (8.90, 11.77)	10.42 (8.70, 12.19)	0.760
22:4n-6 (DTA)	0.49 (0.36, 0.61)	0.47 (0.32, 0.61)	0.440	0.27 (0.22, 0.35)	0.24 (0.20, 0.29)	0.001
22:5n-6 (n6-DPA)	0.53 (0.37, 0.66)	0.51 (0.42, 0.65)	0.940	0.50 (0.35, 0.64)	0.52 (0.39, 0.65)	0.340
Total n-6 PUFAs	37.29 (35.55, 38.71)	37.46 (35.32, 38.67)	0.800	37.21 (35.22, 38.57)	37.34 (35.78, 39.05)	0.170
**PUFA ratios**						
Δ6-desaturase, 18:3n-6/18:2n-6	0.003 (0.003, 0.005)	0.004 (0.003, 0.006)	0.014	0.003 (0.002, 0.005)	0.003 (0.002, 0.004)	0.980
Δ5-desaturase, 20:4n-6/20:3n-6	3.43 (2.61, 4.12)	2.87 (2.30, 3.60)	0.001	2.94 (2.44, 3.82)	2.71 (2.20, 3.36)	0.021
DGLA/LA, 20:3n-6/18:2n-6	0.17 (0.13, 0.20)	0.19 (0.16, 0.23)	<0.001	0.15 (0.13, 0.19)	0.18 (0.14, 0.21)	0.001

^a^Data are presented as median (25th and 75th percentile).

^b^*P* values for differences between case and control participants were obtained by linear mixed models with associated likelihood ratio tests, accounting for matched case–control pairs (likelihood ratio tests).

Abbreviations: AA, arachidonic acid; ALA, alpha-linolenic acid; DGLA, dihomo-gamma-linolenic acid; DHA, docosahexaenoic acid; DPA, docosapentaenoic acid; DTA, docosatetraenoic acid; EDA, eicosadienoic acid; EPA, eicosapentaenoic acid; GDM, gestational diabetes mellitus; GLA, gamma-linolenic acid; LA, linoleic acid; PUFA, polyunsaturated fatty acid

### Longitudinal plasma phospholipid PUFA profile across gestation

Overall, concentrations (%) of plasma phospholipid n-3 PUFA generally decreased, whereas n-6 PUFA fluctuated among both cases and controls as pregnancy progressed (S3 Fig). Case–control differences in n-3 or n-6 PUFAs and PUFA ratios, if any, were mostly observed before diagnosis of GDM (i.e., gestational weeks 24–28) and diminished or even changed the direction in late pregnancy. The only case–control difference was observed in gestational weeks 20–23, with lower PUFA n-3 and higher PUFA n-6 levels in women with GDM than in women without GDM. For the PUFA product-to-precursor ratios, women with GDM had higher levels of Δ6-desaturase and DGLA/AA at gestational weeks 13–15 and 24–27, lower levels of Δ5-desaturase at weeks 13–15 and higher levels at weeks 36–39, and higher n-6/n-3 PUFA ratio at weeks 20–23, respectively (S4 Fig).

### Plasma phospholipid PUFAs in relation to glucose metabolism and cardiometabolic biomarkers

Among plasma phospholipid n-3 PUFAs at gestational weeks 10–14, total n-3 PUFAs and individual DPA and DHA were all inversely correlated with hs-CRP, DHA also positively with adiponectin, and EPA inversely with insulin, HOMA-IR, and triglycerides at weeks 15–26 among non-GDM controls, after adjusting for covariates (Table 3). In a consistent pattern, Δ5-desaturase was inversely correlated with insulin, HOMA-IR, C-peptide, leptin, and triglycerides and positively with HDL-C. In contrast, an opposite pattern was observed for DGLA, with overall positive correlations with biomarkers implicated in glucose intolerance and insulin resistance and inverse correlations with HDL-C. Further, the DGLA/LA ratio was positively correlated with C-peptide and leptin. Similar trends were observed when examining cross-sectional correlations between individual PUFAs and these clinical markers at weeks 10–14, as illustrated in the heat map (S5 Fig).

**Table 3:** Partial Spearman correlation coefficients of plasma phospholipid n-3 PUFA, n-6 PUFA, and PUFA ratios at gestational weeks 10–14 with subsequent fasting plasma cardiometabolic biomarkers and indices at gestational weeks 15–26 among non-GDM controls^a^.

	Glucose	Insulin	HOMA-IR	C-peptide	hs-CRP	HMW adiponectin	Leptin	Total cholesterol	HDL-C	LDL-C	TGs
**n-3 PUFAs**											
18:3n-3 (ALA)	0.01	0.01	0.01	0.03	−0.02	0.11	0.04	0.16	0.03	0.16	0.08
20:5n-3 (EPA)	−0.13	−0.19*	−0.20*	−0.11	0.16	0.14	0.04	−0.01	0.01	0.05	−0.25*
22:5n-3 (DPA)	0.12	0.04	0.04	0.02	−0.23*	−0.09	−0.03	0.02	−0.05	0.08	−0.03
22:6n-3 (DHA)	−0.16	0.01	0.03	−0.03	−0.29^†^	0.19*	−0.08	−0.02	0.07	−0.04	0.01
Total n-3 PUFAs	-0.14	−0.02	0.01	−0.04	−0.30*	0.16	−0.08	−0.02	0.07	−0.03	−0.04
**n-6 PUFAs**											
18:2n-6 (LA)	−0.12	−0.02	−0.02	−0.01	−0.02	0.12	−0.10	0.12	0.06	0.06	0.16
18:3n-6 (GLA)	0.04	0.09	0.09	0.08	0.06	0.03	0.19	−0.04	−0.05	0.04	−0.07
20:2n-6 (EDA)	0.01	0.06	0.07	0.13	0.10	0.04	0.04	0.16	−0.14	0.20*	0.27^‡^
20:3n-6 (DGLA)	0.18*	0.19*	0.20*	0.29^†^	0.16*	−0.04	0.25^†^	−0.03	−0.18*	0.02	0.13
20:4n-6 (AA)	0.12	−0.01	0.01	−0.02	0.01	−0.11	0.08	−0.21*	0.07	−0.22*	−0.22*
22:4n-6 (DTA)	−0.02	−0.02	−0.02	0.08	0.10	0.09	0.06	−0.02	−0.15*	0.07	−0.15*
22:5n-6 (n6-DPA)	−0.08	−0.08	−0.10	−0.08	0.04	−0.01	−0.01	−0.03	−0.08	0.01	−0.03
Total n-6 PUFAs	−0.04	0.01	0.01	0.03	0.03	0.02	−0.01	−0.04	0.09	−0.12	0.06
**Fatty acids ratio**											
Δ6-desaturase, 18:3n-6/18:2n-6	0.06	0.09	0.09	0.07	0.07	−0.02	0.22*	−0.08	−0.07	0.01	−0.10
Δ5-desaturase, 20:4n-6/20:3n-6	−0.11	−0.17*	−0.17*	−0.24*	−0.12	−0.02	−0.18*	−0.05	0.19*	−0.11	−0.18*
DGLA/LA, 20:3n-6/18:2n-6	0.16	0.16	0.16	0.24*	0.15	−0.06	0.26^‡^	−0.04	−0.17	0.03	0.06

^a^Adjusted for age (years), gestational age at blood collection (weeks), parity (nulliparous, multiparous), family history of diabetes (yes, no), and prepregnancy body mass index (<25.0, 25.0–29.9, 30.0–34.9, 35.0–44.9 kg/m^2^).

*P < 0.05 after false-discovery rate correction.

^†^*P* < 0.01 after false-discovery rate correction.

^‡^*P* < 0.001 after false-discovery rate correction.

Abbreviations: AA, arachidonic acid; ALA, alpha-linolenic acid; DGLA, dihomo-gamma-linolenic acid; DHA, docosahexaenoic acid; DPA, docosapentaenoic acid; DTA, docosatetraenoic acid; EDA, eicosadienoic acid; EPA, eicosapentaenoic acid; GDM, gestational diabetes mellitus; GLA, gamma-linolenic acid; HDL-C, high-density lipoprotein cholesterol; HMW, high-molecular-weight; HOMA-IR, homeostasis model assessment of insulin resistance; hs-CRP, high-sensitivity C-reactive protein; LA, linoleic acid; LDL-C, low-density lipoprotein cholesterol; PUFA, polyunsaturated fatty acid; TG, triglyceride

### Plasma phospholipid PUFAs in early to midpregnancy and subsequent GDM risk

**Fig 1:**
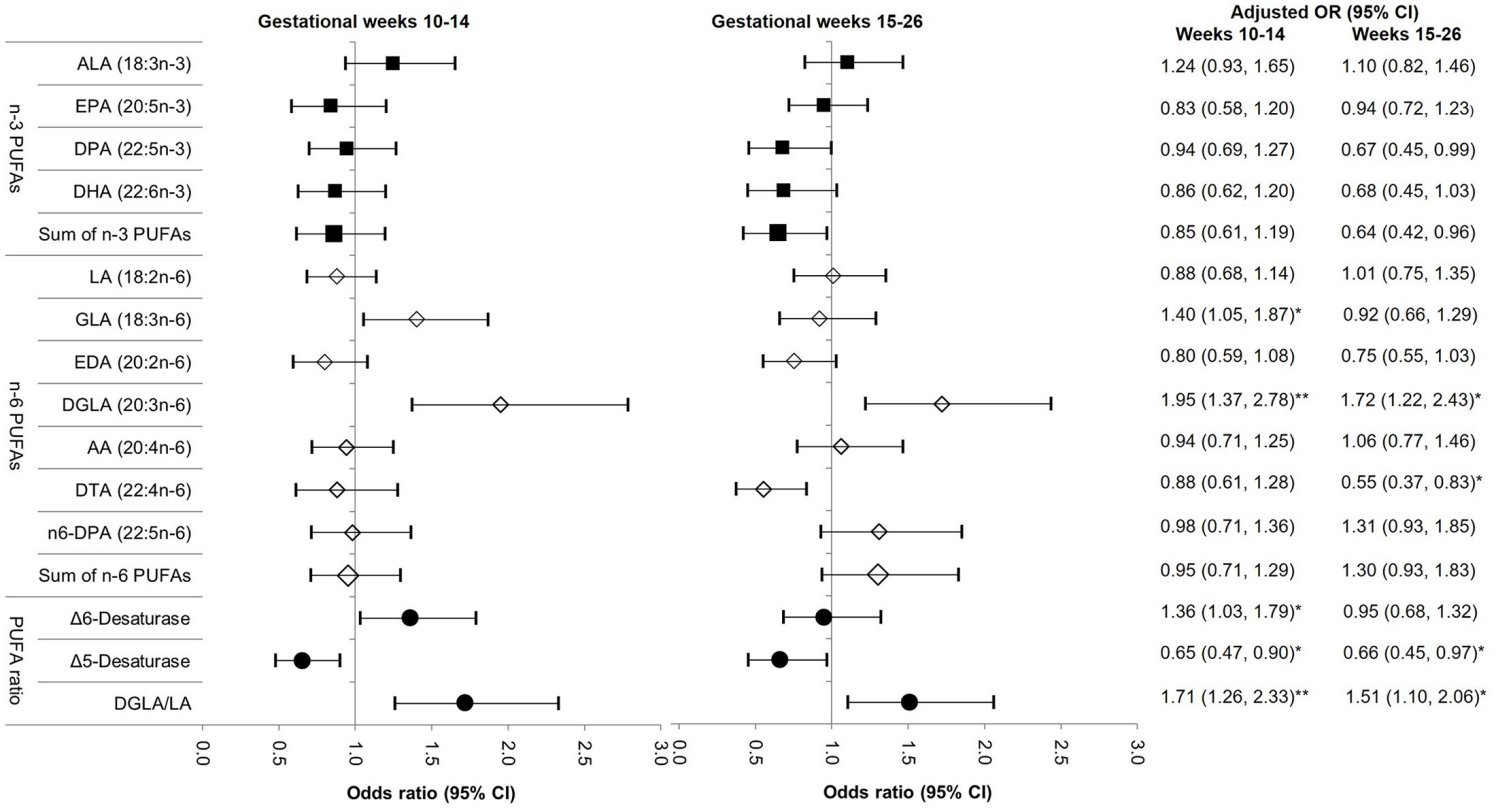
Adjusted OR (95% CIs) of GDM risk per one standard deviation increase in plasma phospholipid n-3 PUFA, n-6 PUFA, and PUFA ratios at gestational weeks 10–14 and 15–26. * *P* <0.05 after false-discovery rate correction ** *P* <0.01 after false-discovery rate correction AA, arachidonic acid; ALA, alpha-linolenic acid; DHA, docosahexaenoic acid; DGLA, dihomo-gamma-linolenic acid; DPA, docosapentaenoic acid; DTA, docosatetraenoic acid; EDA, eicosadienoic acid; EPA, eicosapentaenoic acid; GDM, gestational diabetes mellitus; GLA, gamma-linolenic acid; LA, linoleic acid; OR, odds ratio; PUFA, polyunsaturated fatty acid.

After adjusting for major risk factors for GDM, the individual n-3 DPA and total n-3 PUFAs at gestational weeks 15–26 but not 10–14 were associated with a 33% (adjusted odds ratio [aOR] 0.67; 95% CI 0.45–0.99) and 36% (0.64; 95% CI 0.42–0.96) lower risk of GDM per SD increase, respectively; however, the significance did not persist after post hoc FDR correction (Fig 1; see similar unadjusted results in S6 Fig). Among n-6 PUFAs, total n-6 PUFAs were not significantly associated, whereas individual n-6 PUFAs were differentially associated with GDM risk. Specifically, per SD increase, GLA at weeks 10–14 and DGLA at weeks 10–14 and 15–26 were associated with a 1.40-fold (95% CI 1.05–1.87), 1.95-fold (95% CI 1.37–2.78), and 1.72-fold (95% CI 1.22–2.43) higher risk of GDM, respectively, whereas DTA at weeks 15–26 was associated with a 45% (aOR 0.55; 95% CI 0.37–0.83) lower risk of GDM (all *P* values < 0.05 after FDR correction; Fig 1; see unadjusted results in S6 Fig). For PUFA ratios, Δ5-desaturase at weeks 10–14 and 15–26 was associated with a 35% (0.65; 95% CI 0.47–0.90) and 34% (0.66; 95% CI 0.45–0.97) lower risk of GDM per SD increase, respectively (both *P* values < 0.05 after FDR correction). In contrast, Δ6-desaturase at gestational weeks 10–14 and the DGLA/LA at both gestational periods were significantly related to a 1.36- to 1.71-fold higher risk of GDM per SD increase (all *P* values < 0.05 after FDR correction). In sensitivity analysis additionally adjusting for plasma phospholipid SFAs, we found similar results on individual plasma phospholipid PUFAs except that SFAs attenuated findings of GLA and PUFA ratios from significant to null after FDR correction for *P* values (S1 Table). Further, longitudinal analysis modeling repeated measures of PUFAs at weeks 10–14 and 15–26 showed overall slightly attenuated (due to the averaged-out effect) but consistent patterns with the trimester-specific analysis (S7 Fig). In sensitivity analysis excluding three women with hemoglobin A1C (≥6.5%) at weeks 10–14, an indicator for undiagnosed preexisting diabetes, results remain materially unchanged.

The risk estimates were adjusted for age (years), gestational age at blood collection (weeks), parity (nulliparous, multiparous), family history of diabetes (yes, no), and prepregnancy BMI (<25.0, 25.0–29.9, 30.0–34.9, 35.0–44.9 kg/m^2^).

Results on the longitudinal analysis assessing repeated measures of plasma phospholipid PUFAs across gestational weeks 10–14 and 15–26 in relation to GDM risk are available in S7 Fig.

Consistently, as parameterized in quartiles, individual n-6 DGLA at weeks 10–14 and 15–26 showed positive dose-response relationships with GDM risk (*P* for trend across quartiles after FDR correction = 0.056 and 0.008, respectively; S2 Table), whereas Δ5-desaturase at both gestational periods showed inverse dose-response relationships with GDM risk (*P* for trend after FDR correction = 0.03 and 0.078, respectively), after adjustment for covariates. In stratification analyses, we did not observe significant effect modification by conventional risk factors for GDM, including prepregnancy obesity, family history of diabetes, and race/ethnicity.

**Fig 2:**
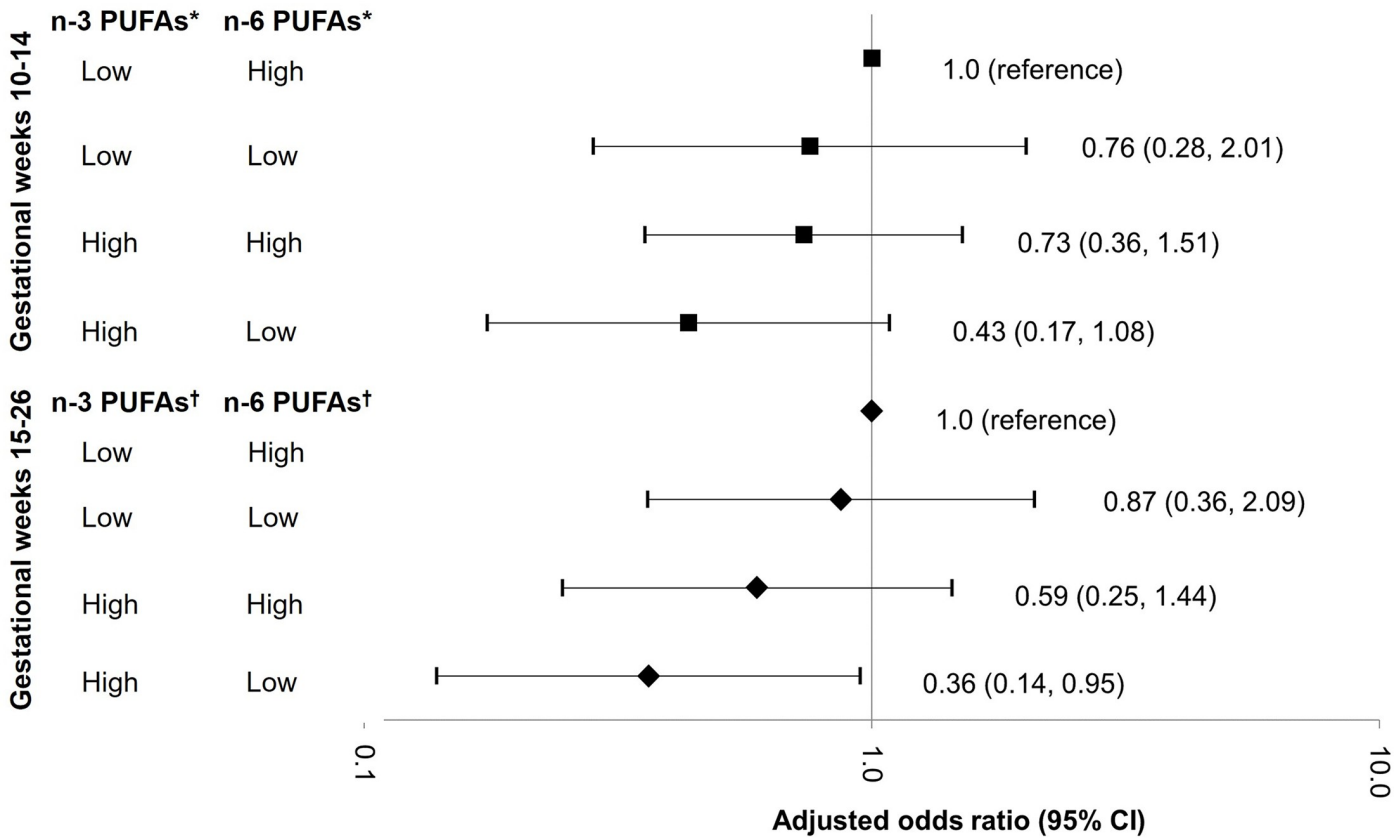
Adjusted odds ratios (95% CIs) of GDM risk in association with plasma phospholipid n-3 PUFA and n-6 PUFA at gestational weeks 10–14 and 15–26. The point estimates were adjusted for age (years), gestational age at blood collection (weeks), parity (nulliparous, multiparous), family history of diabetes (yes, no), and prepregnancy body mass index (<25.0, 25.0–29.9, 30.0–34.9, 35.0–44.9 kg/m^2^). High, concentrations above median; low, concentrations below median. Obtained by the likelihood ratio test for the interaction between n-3 and n-6 PUFAs: *P for interaction = 0.08 at gestational weeks 10–14; ^†^*P* for interaction = 0.02 at gestational weeks 15–26. GDM, gestational diabetes mellitus; PUFA, polyunsaturated fatty acid.

Further, combination of high concentrations (≥median; %) of n-3 PUFAs and low concentrations (<median; %) of n-6 PUFAs illustrated a joint effect on GDM. Compared to women with low n-3 PUFAs and high n-6 PUFAs, those with high n-3 PUFAs and low n-6 PUFAs levels at gestational weeks 15–26 were at a 64% (aOR 0.36; 95% CI 0.14–0.95; *P* for interaction = 0.02) lower risk of GDM (Fig 2). An inverse but not statistically significant association was observed for the combination of high n-3 PUFAs and low n-6 PUFAs at weeks 10–14 (0.43; 95% CI 0.17–1.08; *P* for interaction = 0.08).

## Discussion

In this longitudinal study within the prospective NICHD Fetal Growth Studies–Singleton Cohort, we provide, to our knowledge, the most extensive evaluation to date of the longitudinal profile of 11 individual plasma phospholipid PUFAs and three PUFA product-to-precursor ratios across gestation and their associations in early to midpregnancy with subsequent risk of GDM. Overall, primarily diet-derived plasma phospholipid PUFAs including n-3 EPA and DHA and n-6 LA in early to midpregnancy were not significantly associated with GDM risk; however, EPA and DHA were inversely correlated with insulin-resistance markers. Among primarily endogenous PUFAs, n-6 GLA at weeks 10–14 and DGLA at both weeks 10–14 and 15–26 were positively associated with GDM risk, whereas DTA at weeks 15–26 was inversely related to GDM risk. For PUFA ratios, Δ6-desaturase at weeks 10–14 and DGLA/LA at both gestational periods were positively associated whereas Δ5-desaturase at both gestational periods was inversely associated with GDM risk.

### Comparison with other studies on GDM

Previous data on plasma phospholipid PUFAs in relation to risk of GDM were scarce and largely based on small-scaled retrospective or cross-sectional studies with one point-in-time assessment of PUFAs profiles at or after the diagnosis of GDM or even after delivery, including 12–62 GDM cases [39–45], whereas only one previous study measured serum PUFAs before GDM diagnosis [24]. The limited data and inadequate design to address the prospective association may be attributed to the unique challenges including but not limited to temporal examination of exposure and outcome of interest, participant burden due to multiple measurements, and cohort retention among pregnant populations. Given that PUFA profiles may be affected by GDM treatment, assessment concurrent with or after GDM diagnosis could not adequately inform the pathophysiology of GDM. Therefore, we compared our study with the only existing prospective study by Chen and colleagues with a small sample size (49 GDM cases) [24]. Consistent with our findings, Chen and colleagues reported null associations of three serum n-3 (ALA, EPA, DHA) and two n-6 PUFAs (LA and AA) at gestational week 16.5 with GDM risk but no data on other individual PUFAs. The null association of DHA with GDM in observational studies is consistent with one randomized controlled trial, which found no reduction in GDM risk by DHA supplementation in the second half of pregnancy [46]. On the other hand, although our findings demonstrate null associations of EPA and DHA with GDM risk, they do not exclude the possibility of benefit on glucose–insulin homeostasis based on their inverse correlations with insulin, HOMA-IR (both with EPA), and hs-CRP (with DHA) and positive correlation with adiponectin (with DHA). Further, the overall downward trend of plasma phospholipid n-3 PUFA across gestation in our study was consistent with previous data showing progressive decrease in plasma n-3 PUFAs, suggesting increasing maternal–fetal transfer due to the increasing demand of fetal development [25]. Given the circulating levels of n-3 PUFAs are a function of both exogenous and endogenous sources, future studies investigating relative contributions of exogenous (e.g., diet, physical activity, smoking) and endogenous factors (e.g., genetics and biochemistry) to circulating levels of n-3 PUFAs during pregnancy are warranted.

### Comparison with other studies on T2DM

Our findings are in line with some, though not all, previous data on T2DM among nonpregnant individuals. For instance, plasma phospholipid DTA was inversely and GLA and DGLA were positively associated with T2DM risk [34], whereas heterogeneous and inconclusive associations of plasma phospholipid n-3 PUFAs with T2DM have been reported in a recent meta-analysis [14]. Among n-6 PUFAs, Forouhi and colleagues reported a strong inverse association of T2DM with the most abundant plasma phospholipid LA [34] and a meta-analysis reported reduction in fasting insulin and HOMA-IR by plant-derived PUFAs (primarily LA) among people without diabetes [18], in contrast to a positive association of dietary LA with T2DM risk [47] and to our findings of null associations of LA in early to midpregnancy with GDM risk. It is plausible that the differential findings may be attributed to unique metabolic status of pregnancy because of the increasing demand of fetal requirement of essential PUFAs and thus potentially different mechanisms underlying plasma phospholipid PUFAs and GDM versus T2DM. Future investigation is needed to dissect the various mechanisms underlying GDM and its evolution to T2DM after pregnancy. Notably, the plasma phospholipid PUFA compositions in our study were comparable to those in nonpregnant populations [34,48], with the only exception for an approximately 10-fold difference between DHA and EPA in our study (4.0% versus 0.3%). This magnitude of difference was similar to that observed in other pregnant populations [49] but greater than that in nonpregnant individuals (e.g., 4% versus 1%) [34,48], which could be partially explained by greater consumption of seafood and/or prenatal supplements rich in DHA among pregnant women [50,51]. This observation further demonstrates the unique profile of plasma phospholipid PUFAs among pregnant women and cautions against simple extrapolation from data on nonpregnant populations.

### Biological plausibility and implications

Although the exact metabolic pathways whereby plasma phospholipid PUFAs are involved in glucose homeostasis and the development of GDM remain to be elucidated, our findings are biologically plausible. Plasma phospholipid PUFAs may alter cell membrane structure and property, influence the response of membrane-bound hormone receptors, serve as precursors to proinflammatory eicosanoids, and affect downstream insulin and glucose metabolism [52].

Consistent with our observations of inverse associations of total n-3 PUFAs and individual EPA, DPA, and DHA with several insulin-resistance markers, animal data have shown that n-3 PUFAs may decrease secretion of inflammatory cytokines and reverse glucose intolerance [53,54]. Among plasma phospholipid n-6 PUFAs, individual GLA and DGLA, intermediate metabolites of AA, were positively associated with GDM risk in our study. Animal studies demonstrated the ability of predominantly endogenous GLA and DGLA to modulate cellular lipid metabolism and eicosanoid synthesis [55], which in turn are implicated in inflammatory-induced insulin resistance and β-cell destruction [56]. Further, our finding of an inverse association of Δ5-desaturase (DGLA to AA) with GDM risk was consistent with an inverse causal relation of Δ5-desaturase activity encoded by the fatty acid desaturase 1 (*FADS1*) with diabetes risk in a mendelian randomization study [57]. On the other hand, we did not observe a significant association between plasma phospholipid LA, the most abundant form of circulating PUFAs that is essentially diet-derived, with risk of GDM. Health effects of LA remain debated, even with uncertainties in dietary recommendations. American Heart Association supports an n-6 PUFA (primarily LA) intake of 5%–10% of total energy [58], whereas the French Food Safety Agency recommends LA < 4% of energy to avoid potential harm [59]. The theorized harm is mainly rooted in LA as the precursor to AA. However, previous data suggest that dietary intake of LA has little effect on circulating AA and its metabolites GLA and DGLA, suggesting a stronger role of endogenous regulation of these metabolites [60]. Our findings of null associations of LA with markers of glucose homeostasis and GDM risk do not suggest a harmful role of circulating LA in GDM pathophysiology. Notably, sensitivity analysis showed that the significant associations of PUFA ratios with GDM risk were attenuated and turned null after further adjustment for plasma phospholipid SFAs and FDR correction for *P* values, suggesting the potential interplay and metabolic effects of different fatty acid components [61]. Future investigations on the role of complex interplay among fatty acids subclasses in glucose homeostasis and GDM risk are warranted.

### Strengths and limitations

Our study has some notable strengths. The prospective and longitudinal data collection allowed examination of the temporal associations of PUFAs during early to midpregnancy with subsequent GDM risk. Further, we had the unique ability to profile the longitudinal physiologic trends of plasma phospholipid PUFAs throughout pregnancy, which demonstrated differential temporal variations of PUFA composition and desaturase activity among women with and without GDM. Most notably, the objective measurement of plasma phospholipid PUFA levels enabled assessment of GDM risk with individual and subclasses of circulating PUFAs. Therefore, our findings may shed light on previous inconsistent inferences concerning dietary intakes of PUFAs in relation to glucose homeostasis and diabetes risk, which has been inevitably subject to measurement errors of dietary assessment via subjective report [11–14]. Moreover, we measured a comprehensive panel of markers of glucose metabolism and cardiometabolic risk simultaneously with plasma phospholipid PUFAs, which may provide mechanistic insight into the association of circulating PUFAs with risk of GDM.

Some potential limitations of our study merit discussion. Concentrations of individual plasma phospholipid PUFAs were measured as relative (percent of total phospholipid fatty acids), not absolute, concentrations. However, this approach has been validated and widely adopted in epidemiological studies, which tends to facilitate a better interpretation of metabolic associations compared to absolute measurements [62]. In the present study, we did not assess the associations of specific fractions of plasma phospholipid PUFAs (e.g., phosphatidylcholine, phosphatidylserine, phosphatidylethanolamine, etc.) with risk of GDM, which warrant examination in future studies. Fasting plasma samples were collected at gestational weeks 15–26 and random samples at other visits. However, the fasting duration prior to biospecimen collection at all visits were nondifferential to the case–control status; thus, differential measurement error due to fasting status is unlikely. Given the clinically meaningful competition between n-3 and n-6 PUFAs, we assessed the joint associations of n-3 and n-6 PUFAs categories with GDM risk while also acknowledging the categorization is data-driven given the lack of established reference range or threshold values. Despite the significant associations observed between individual PUFAs and GDM risk, we cannot exclude the possibility of the relatively modest sample size causing underestimation of the significance of true associations due to statistical power. To date, our study is one of the largest longitudinal studies of plasma phospholipid PUFAs throughout pregnancy in relation to GDM risk; future studies with a larger sample size are warranted to validate our findings. The generalization of our findings to obese women with otherwise low-risk obstetrical profiles remains to be established; however, inclusion of overall healthy women may minimize reverse causality and the residual confounding due to preexisting complications and unhealthy behaviors. Finally, our findings are based on an observational study within a prospective cohort of pregnant women; further intervention studies are warranted to confirm our findings and the causal relationship.

### Conclusions

In this longitudinal study, our findings of differential associations of primarily endogenously metabolized n-6 PUFAs including GLA, DGLA, and DTA with GDM risk may suggest a potential role of circulating levels of these individual phospholipid PUFAs in early to midpregnancy in GDM pathophysiology. Among primarily diet-derived PUFAs, our findings do not provide strong evidence to suggest beneficial roles of plasma phospholipid n-3 EPA and DHA in the prevention of GDM, although not excluding the possibility of benefit on glucose homeostasis given their inverse correlations with insulin-resistance markers. Further, null findings on the plant-derived n-6 LA suggest neither a harmful nor a beneficial role in GDM pathophysiology. Collectively, these findings highlight the need to recognize the distinct associations between individual plasma phospholipid PUFAs and GDM risk, rather than merely focusing on dietary assessment of total or subclasses of PUFAs, given the various processes and interplay in the biosynthesis pathways of individual PUFAs. Preventive strategies to mitigate GDM risk could be potentially strengthened by considering the differential glucose homeostasis and cardiometabolic effects varying by subclasses of PUFAs and individual circulating PUFAs.

## Supporting information

S1 STROBE Checklist (PDF)

S1 Protocol Study protocol. (PDF)

S1 Fig Flow chart of the nested case–control study, within the NICHD Fetal Growth Studies–Singleton Cohort. NICHD, National Institute of Child Health and Human Development.
(PDF)

S2 Fig Metabolic pathways and major exogenous (dietary) and endogenous (lipogenesis) sources of polyunsaturated fatty acids. (PDF)

S3 Fig Longitudinal profiles (mean ± standard errors, %) of plasma phospholipid n-3 PUFA and n-6 PUFA throughout pregnancy according to gestational-age intervals among women with and without GDM. GDM, gestational diabetes mellitus; PUFA, polyunsaturated fatty acid.
(PDF)

S4 Fig Longitudinal profiles (mean ± standard errors, %) of plasma phospholipid PUFA ratios throughout pregnancy according to gestational-age intervals among women with and without GDM. GDM, gestational diabetes mellitus; PUFA, polyunsaturated fatty acid.
(PDF)

S5 Fig Heat map of the correlation matrix of plasma phospholipid n-3 PUFA, n-6 PUFA, and PUFA ratios with glucose metabolism and cardiometabolic markers at gestational weeks 10–14 among non-GDM controls. GDM, gestational diabetes mellitus; PUFA, polyunsaturated fatty acid.
(PDF)

S6 Fig Unadjusted odds ratios (95% CIs) of GDM risk per one standard deviation increase in plasma phospholipid n-3 PUFA, n-6 PUFA, and PUFA ratios at gestational weeks 10–14 and 15–26. GDM, gestational diabetes mellitus; PUFA, polyunsaturated fatty acid.
(PDF)

S7 Fig Adjusted odds ratios (95% CIs) of GDM risk in association with longitudinal changes of plasma phospholipid n-3 PUFA, n-6 PUFA, and PUFA ratios per one standard deviation increase from gestational weeks 10–14 to 15–26. GDM, gestational diabetes mellitus; PUFA, polyunsaturated fatty acid.
(PDF)

S1 Table Sensitivity analyses for the association of plasma phospholipid n-3 PUFA, n-6 PUFA, and PUFA ratios at gestational weeks 10–14 and 15–26 with GDM risk. GDM, gestational diabetes mellitus; PUFA, polyunsaturated fatty acid.
(PDF)

S2 Table Odds ratios (95% CIs) for subsequent risk of GDM according to quartiles of plasma phospholipid n-3 PUFA, n-6 PUFA, and PUFA ratios at gestational weeks 10–14 and 15–26. GDM, gestational diabetes mellitus; PUFA, polyunsaturated fatty acid.
(PDF)

## Abbreviations

AA: arachidonic acid
ALA: alpha-linolenic acid
aOR: adjusted odds ratio
BMI: body mass index
CV: coefficient of variation
DGLA: dihomo-gamma-linolenic acid
DHA: docosahexaenoic acid
DPA: docosapentaenoic acid
DTA: docosatetraenoic acid
EDA: eicosadienoic acid
EPA: eicosapentaenoic acid
FADS1: fatty acid desaturase 1
FDR: false discovery rate
GDM: gestational diabetes mellitus
GLA: gamma-linolenic acid
HDL-C: high-density lipoprotein cholesterol
HMW: high-molecular-weight
HOMA-IR: homeostasis model assessment of insulin resistance
hs-CRP: high-sensitivity C-reactive protein
IQR: interquartile range
LA: linoleic acid
LDL-C: low-density lipoprotein cholesterol
NICHD: National Institute of Child Health and Human Development
OR: odds ratio
PUFA: polyunsaturated fatty acid
SD: standard deviation
SFA: saturated fatty acid
T2DM: type 2 diabetes mellitus

## References

[1] Guariguata L, Linnenkamp U, Beagley J, Whiting DR, Cho NH. Global estimates of the prevalence of hyperglycaemia in pregnancy. Diabetes research and clinical practice. 2014;103(2):176–85. 10.1016/j.diabres.2013.11.003 24300020

[2] Ferrara A. Increasing prevalence of gestational diabetes mellitus: a public health perspective. Diabetes Care. 2007;30 Suppl 2:S141–6. 10.2337/dc07-s206 . 17596462

[3] Zhu Y, Zhang C. Prevalence of Gestational Diabetes and Risk of Progression to Type 2 Diabetes: a Global Perspective. Curr Diab Rep. 2016;16(1):7 10.1007/s11892-015-0699-x . 26742932 PMC6675405

[4] Duque-Guimarães DE, Ozanne SE. Nutritional programming of insulin resistance: causes and consequences. Trends in Endocrinology & Metabolism. 2013;24(10):525–35. 23791137 10.1016/j.tem.2013.05.006

[5] Bantle JP, Laine DC, Castle GW, Thomas JW, Hoogwerf BJ, Goetz FC. Postprandial glucose and insulin responses to meals containing different carbohydrates in normal and diabetic subjects. New Engl J Med. 1983;309(1):7–12. 10.1056/NEJM198307073090102 6343873

[6] Boden G, She P, Mozzoli M, Cheung P, Gumireddy K, Reddy P, et al Free fatty acids produce insulin resistance and activate the proinflammatory nuclear factor-κB pathway in rat liver. Diabetes. 2005;54(12):3458–65. 10.2337/diabetes.54.12.3458 16306362

[7] Koves TR, Ussher JR, Noland RC, Slentz D, Mosedale M, Ilkayeva O, et al Mitochondrial overload and incomplete fatty acid oxidation contribute to skeletal muscle insulin resistance. Cell metabolism. 2008;7(1):45–56. 10.1016/j.cmet.2007.10.013 18177724

[8] Storlien LH, Jenkins AB, Chisholm DJ, Pascoe WS, Khouri S, Kraegen EW. Influence of dietary fat composition on development of insulin resistance in rats: relationship to muscle triglyceride and ω-3 fatty acids in muscle phospholipid. Diabetes. 1991;40(2):280–9. 10.2337/diab.40.2.280 1991575

[9] United States Department of Health Human Services. Dietary Guidelines for Americans 2015–2020. Washington, DC: Skyhorse Publishing; 2017.

[10] Eyre H, Kahn R, Robertson RM, Clark NG, Doyle C, Hong Y, et al Preventing cancer, cardiovascular disease, and diabetes: a common agenda for the American Cancer Society, the American Diabetes Association, and the American Heart Association. Circulation. 2004;109(25):3244–55. 10.1161/01.CIR.0000133321.00456.00 . 15198946

[11] Akinkuolie AO, Ngwa JS, Meigs JB, Djousse L. Omega-3 polyunsaturated fatty acid and insulin sensitivity: a meta-analysis of randomized controlled trials. Clinical nutrition. 2011;30(6):702–7. 10.1016/j.clnu.2011.08.013 . 21959352 PMC5066815

[12] Hartweg J, Perera R, Montori V, Dinneen S, Neil HA, Farmer A. Omega-3 polyunsaturated fatty acids (PUFA) for type 2 diabetes mellitus. Cochrane Database Syst Rev. 2008;(1):Cd003205. 10.1002/14651858.CD003205.pub2 . 18254017 PMC9006221

[13] Wallin A, Di Giuseppe D, Orsini N, Patel PS, Forouhi NG, Wolk A. Fish consumption, dietary long-chain n-3 fatty acids, and risk of type 2 diabetes: systematic review and meta-analysis of prospective studies. Diabetes Care. 2012;35(4):918–29. 10.2337/dc11-1631 . 22442397 PMC3308304

[14] Wu JH, Micha R, Imamura F, Pan A, Biggs ML, Ajaz O, et al Omega-3 fatty acids and incident type 2 diabetes: a systematic review and meta-analysis. The British journal of nutrition. 2012;107 Suppl 2:S214–27. 10.1017/s0007114512001602 . 22591895 PMC3744862

[15] Zheng JS, Huang T, Yang J, Fu YQ, Li D. Marine N-3 polyunsaturated fatty acids are inversely associated with risk of type 2 diabetes in Asians: a systematic review and meta-analysis. PLoS ONE. 2012;7(9):e44525 10.1371/journal.pone.0044525 . 22984522 PMC3439396

[16] Xun P, He K. Fish Consumption and Incidence of Diabetes: meta-analysis of data from 438,000 individuals in 12 independent prospective cohorts with an average 11-year follow-up. Diabetes Care. 2012;35(4):930–8. 10.2337/dc11-1869 . 22442398 PMC3308299

[17] Imamura F, Micha R, Wu JH, de Oliveira Otto MC, Otite FO, Abioye AI, et al Effects of Saturated Fat, Polyunsaturated Fat, Monounsaturated Fat, and Carbohydrate on Glucose-Insulin Homeostasis: A Systematic Review and Meta-analysis of Randomised Controlled Feeding Trials. PLoS Med. 2016;13(7):e1002087 10.1371/journal.pmed.1002087 . 27434027 PMC4951141

[18] Wanders AJ, Blom WA, Zock PL, Geleijnse JM, Brouwer IA, Alssema M. Plant-derived polyunsaturated fatty acids and markers of glucose metabolism and insulin resistance: a meta-analysis of randomized controlled feeding trials. BMJ Open Diabetes Research and Care. 2019;7(1):e000585 10.1136/bmjdrc-2018-000585 30899527 PMC6398820

[19] Wu JHY, Marklund M, Imamura F, Tintle N, Ardisson Korat AV, de Goede J, et al Omega-6 fatty acid biomarkers and incident type 2 diabetes: pooled analysis of individual-level data for 39 740 adults from 20 prospective cohort studies. The lancet Diabetes & endocrinology. 2017;5(12):965–74. 10.1016/s2213-8587(17)30307-8 . 29032079 PMC6029721

[20] Chowdhury R, Warnakula S, Kunutsor S, Crowe F, Ward HA, Johnson L, et al Association of dietary, circulating, and supplement fatty acids with coronary risk: a systematic review and meta-analysis. Ann Intern Med. 2014;160(6):398–406. 10.7326/M13-1788 . 24723079

[21] Ramsden CE, Zamora D, Leelarthaepin B, Majchrzak-Hong SF, Faurot KR, Suchindran CM, et al Use of dietary linoleic acid for secondary prevention of coronary heart disease and death: evaluation of recovered data from the Sydney Diet Heart Study and updated meta-analysis. Bmj. 2013;346:e8707 10.1136/bmj.e8707 . 23386268 PMC4688426

[22] Farvid MS, Ding M, Pan A, Sun Q, Chiuve SE, Steffen LM, et al Dietary linoleic acid and risk of coronary heart disease: a systematic review and meta-analysis of prospective cohort studies. Circulation. 2014;130(18):1568–78. 10.1161/CIRCULATIONAHA.114.010236 25161045 PMC4334131

[23] Sprecher H, Luthria DL, Mohammed B, Baykousheva SP. Reevaluation of the pathways for the biosynthesis of polyunsaturated fatty acids. Journal of lipid research. 1995;36(12):2471–7. 8847474

[24] Chen X, Scholl TO, Leskiw M, Savaille J, Stein TP. Differences in maternal circulating fatty acid composition and dietary fat intake in women with gestational diabetes mellitus or mild gestational hyperglycemia. Diabetes Care. 2010;33(9):2049–54. 10.2337/dc10-0693 . 20805277 PMC2928361

[25] Schwarz KB. Progressive decrease in plasma omega 3 and omega 6 fatty acids during pregnancy: time course and effects of dietary fats and antioxidant nutrients. Journal of nutritional & environmental medicine. 1998;8(4):335–44.

[26] Buck Louis GM, Grewal J, Albert PS, Sciscione A, Wing DA, Grobman WA, et al Racial/ethnic standards for fetal growth: the NICHD Fetal Growth Studies. Am J Obstet Gynecol. 2015;213(4):449 e1–e41. 10.1016/j.ajog.2015.08.032 . 26410205 PMC4584427

[27] American College of Obstetricians and Gynecologists Committee on Practice Bulletins—Obstetrics. Practice Bulletin No. 137: Gestational diabetes mellitus. Clinical management guidelines for obstetrician-gynecologists. Obstet Gynecol. 2013;122(2 Pt 1):406–16. 10.1097/01.AOG.0000433006.09219.f1 23969827

[28] Zhu Y, Mendola P, Albert PS, Bao W, Hinkle SN, Tsai MY, et al Insulin-like growth factor axis and gestational diabetes: A longitudinal study in a multiracial cohort. Diabetes. 2016 10.2337/db16-0514 PMC5079637 27468747

[29] Levine RJ, Maynard SE, Qian C, Lim KH, England LJ, Yu KF, et al Circulating angiogenic factors and the risk of preeclampsia. New Engl J Med. 2004;350(7):672–83. 10.1056/NEJMoa031884 14764923

[30] Levine RJ, Thadhani R, Qian C, Lam C, Lim KH, Yu KF, et al Urinary placental growth factor and risk of preeclampsia. JAMA. 2005;293(1):77–85. 10.1001/jama.293.1.77 . 15632339

[31] Cao J, Schwichtenberg KA, Hanson NQ, Tsai MY. Incorporation and clearance of omega-3 fatty acids in erythrocyte membranes and plasma phospholipids. Clinical chemistry. 2006;52(12):2265–72. 10.1373/clinchem.2006.072322 . 17053155

[32] Muthayya S, Dwarkanath P, Thomas T, Ramprakash S, Mehra R, Mhaskar A, et al The effect of fish and omega-3 LCPUFA intake on low birth weight in Indian pregnant women. European journal of clinical nutrition. 2009;63(3):340–6. 10.1038/sj.ejcn.1602933 . 17957193

[33] Dirix C. The functionality of maternal and neonatal fatty acids: from pregnancy to childhood [doctoral thesis]. Maastricht, the Netherlands: Maastricht University; 2009.

[34] Forouhi NG, Imamura F, Sharp SJ, Koulman A, Schulze MB, Zheng J, et al Association of Plasma Phospholipid n-3 and n-6 Polyunsaturated Fatty Acids with Type 2 Diabetes: The EPIC-InterAct Case-Cohort Study. PLoS Med. 2016;13(7):e1002094 10.1371/journal.pmed.1002094 . 27434045 PMC4951144

[35] Vessby B, Gustafsson IB, Tengblad S, Boberg M, Andersson A. Desaturation and elongation of fatty acids and insulin action. Annals of the New York Academy of Sciences. 2002;967(1):183–95. 12079847 10.1111/j.1749-6632.2002.tb04275.x

[36] Friedewald WT, Levy RI, Fredrickson DS. Estimation of the concentration of low-density lipoprotein cholesterol in plasma, without use of the preparative ultracentrifuge. Clinical chemistry. 1972;18(6):499–502. . 4337382

[37] Matthews DR, Hosker JP, Rudenski AS, Naylor BA, Treacher DF, Turner RC. Homeostasis model assessment: insulin resistance and beta-cell function from fasting plasma glucose and insulin concentrations in man. Diabetologia. 1985;28(7):412–9. 10.1007/bf00280883 . 3899825

[38] Benjamini Y, Hochberg Y. Controlling the False Discovery Rate: A Practical and Powerful Approach to Multiple Testing. Journal of the Royal Statistical Society Series B (Methodological). 1995;57(1):289–300.

[39] Burlina S, Dalfrà M, Barison A, Marin R, Ragazzi E, Sartore G, et al Plasma phospholipid fatty acid composition and desaturase activity in women with gestational diabetes mellitus before and after delivery. Acta diabetologica. 2017;54(1):45–51. 10.1007/s00592-016-0901-x 27638302

[40] Loosemore ED, Judge MP, Lammi-Keefe CJ. Dietary intake of essential and long-chain polyunsaturated fatty acids in pregnancy. Lipids. 2004;39(5):421–4. 10.1007/s11745-004-1246-y . 15506236

[41] Min Y, Ghebremeskel K, Lowy C, Thomas B, Crawford MA. Adverse effect of obesity on red cell membrane arachidonic and docosahexaenoic acids in gestational diabetes. Diabetologia. 2004;47(1):75–81. 10.1007/s00125-003-1275-5 14634727

[42] Min Y, Nam J-H, Ghebremeskel K, Kim A, Crawford M. A distinctive fatty acid profile in circulating lipids of Korean gestational diabetics: A pilot study. Diabetes research and clinical practice. 2006;73(2):178–83. 10.1016/j.diabres.2006.01.003. 16455150

[43] Thomas B, Ghebremeskel K, Lowy C, Min Y, Crawford MA. Plasma AA and DHA levels are not compromised in newly diagnosed gestational diabetic women. European journal of clinical nutrition. 2004;58(11):1492–7. 10.1038/sj.ejcn.1601996 . 15162132

[44] Wijendran V, Bendel RB, Couch SC, Philipson EH, Thomsen K, Zhang X, et al Maternal plasma phospholipid polyunsaturated fatty acids in pregnancy with and without gestational diabetes mellitus: relations with maternal factors. Am J Clin Nutr. 1999;70(1):53–61. Epub 1999/07/07. 10.1093/ajcn/70.1.53 . 10393139

[45] Fugmann M, Uhl O, Hellmuth C, Hetterich H, Kammer NN, Ferrari U, et al Differences in the serum nonesterified fatty acid profile of young women associated with a recent history of gestational diabetes and overweight/obesity. PLoS ONE. 2015;10(5):e0128001 10.1371/journal.pone.0128001 26011768 PMC4444334

[46] Zhou SJ, Yelland L, McPhee AJ, Quinlivan J, Gibson RA, Makrides M. Fish-oil supplementation in pregnancy does not reduce the risk of gestational diabetes or preeclampsia. Am J Clin Nutr. 2012;95(6):1378–84. 10.3945/ajcn.111.033217 . 22552037

[47] Hodge AM, English DR, O’Dea K, Sinclair AJ, Makrides M, Gibson RA, et al Plasma phospholipid and dietary fatty acids as predictors of type 2 diabetes: interpreting the role of linoleic acid. Am J Clin Nutr. 2007;86(1):189–97. 10.1093/ajcn/86.1.189 . 17616780

[48] Mozaffarian D, Lemaitre RN, King IB, Song X, Huang H, Sacks FM, et al Plasma phospholipid long-chain ω-3 fatty acids and total and cause-specific mortality in older adults: a cohort study. Annals of internal medicine. 2013;158(7):515–25. 10.7326/0003-4819-158-7-201304020-00003 23546563 PMC3698844

[49] Wilson NA, Mantzioris E, Middleton PT, Muhlhausler BS. Gestational age and maternal status of DHA and other polyunsaturated fatty acids in pregnancy: A systematic review. Prostaglandins Leukot Essent Fatty Acids. 2019;144:16–31. 10.1016/j.plefa.2019.04.006 . 31088623

[50] National Institutes of Health. Dietary Supplement Label Database. 2015 [cited 2019 May 24]. http://www.dsld.nlm.nih.gov/dsld/.

[51] U.S. Department of Agriculture Agricultural Research Service. USDA National Nutrient Database for Standard Reference Legacy Release. 2018 Apr [cited 2019 May 24]. https://ndb.nal.usda.gov/ndb/.

[52] Borkman M, Storlien LH, Pan DA, Jenkins AB, Chisholm DJ, Campbell LV. The relation between insulin sensitivity and the fatty-acid composition of skeletal-muscle phospholipids. The New England journal of medicine. 1993;328(4):238–44. 10.1056/NEJM199301283280404 . 8418404

[53] Lamping KG, Nuno DW, Coppey LJ, Holmes AJ, Hu S, Oltman CL, et al Modification of high saturated fat diet with n-3 polyunsaturated fat improves glucose intolerance and vascular dysfunction. Diabetes, obesity & metabolism. 2013;15(2):144–52. 10.1111/dom.12004 . 22950668 PMC3674571

[54] Pahlavani M, Ramalho T, Koboziev I, LeMieux MJ, Jayarathne S, Ramalingam L, et al Adipose tissue inflammation in insulin resistance: review of mechanisms mediating anti-inflammatory effects of omega-3 polyunsaturated fatty acids. J Invest Med. 2017;65(7):1021–7. 10.1136/jim-2017-000535 PMC7561007 28954844

[55] Wang X, Lin H, Gu Y. Multiple roles of dihomo-γ-linolenic acid against proliferation diseases. Lipids in health and disease. 2012;11(1):25. 22333072 10.1186/1476-511X-11-25 PMC3295719

[56] Luo P, Wang M-H. Eicosanoids, β-cell function, and diabetes. Prostaglandins & other lipid mediators. 2011;95(1–4):1–10. 21757024 10.1016/j.prostaglandins.2011.06.001 PMC3144311

[57] Kroger J, Zietemann V, Enzenbach C, Weikert C, Jansen EH, Doring F, et al Erythrocyte membrane phospholipid fatty acids, desaturase activity, and dietary fatty acids in relation to risk of type 2 diabetes in the European Prospective Investigation into Cancer and Nutrition (EPIC)-Potsdam Study. Am J Clin Nutr. 2011;93(1):127–42. 10.3945/ajcn.110.005447 . 20980488

[58] Harris WS, Mozaffarian D, Rimm E, Kris-Etherton P, Rudel LL, Appel LJ, et al Omega-6 fatty acids and risk for cardiovascular disease: a science advisory from the American Heart Association Nutrition Subcommittee of the Council on Nutrition, Physical Activity, and Metabolism; Council on Cardiovascular Nursing; and Council on Epidemiology and Prevention. Circulation. 2009;119(6):902–7. 10.1161/CIRCULATIONAHA.108.191627 19171857

[59] French Food Safety Agency. Opinion of the French Food Safety Agency on the update of French population reference intakes (ANCs) for fatty acids. 2010.

[60] Rett BS, Whelan J. Increasing dietary linoleic acid does not increase tissue arachidonic acid content in adults consuming Western-type diets: a systematic review. Nutrition & metabolism. 2011;8(1):36. 21663641 10.1186/1743-7075-8-36 PMC3132704

[61] Zhu Y, Tsai MY, Sun Q, Hinkle SN, Rawal S, Mendola P, et al A prospective and longitudinal study of plasma phospholipid saturated fatty acid profile in relation to cardiometabolic biomarkers and the risk of gestational diabetes. Am J Clin Nutr. 2018;107(6):1017–26. 10.1093/ajcn/nqy051 . 29868913 PMC6248709

[62] Hodson L, Skeaff CM, Fielding BA. Fatty acid composition of adipose tissue and blood in humans and its use as a biomarker of dietary intake. Prog Lipid Res. 2008;47(5):348–80. 10.1016/j.plipres.2008.03.003 . 18435934

