# Supplementary material for: Plasma phospholipid n-3 and n-6 polyunsaturated fatty acids in relation to cardiometabolic markers and gestational diabetes: A longitudinal study within the prospective NICHD Fetal Growth Studies
Source: PLoS Med. 2019 Sep 13;16(9):e1002910. doi: 10.1371/journal.pmed.1002910 (PMC6743768; doi:10.1371/journal.pmed.1002910)
Supplement: S1 STROBE Checklist — (PDF) [file pmed.1002910.s001.pdf]

STROBE Statement—checklist of items that should be included in reports of observational studies

	Item No	Recommendation	Section	Paragraph number in each section
**Title and abstract**	1	(*a*) Indicate the study's design with a commonly used term in the title or the abstract	Title; Abstract-Background	1
		(*b*) Provide in the abstract an informative and balanced summary of what was done and what was found	Abstract	1-3
**Introduction**				
Background/rationale	2	Explain the scientific background and rationale for the investigation being reported	Introduction	1-2
Objectives	3	State specific objectives, including any prespecified hypotheses	Introduction	3
**Methods**				
Study design	4	Present key elements of study design early in the paper	Methods - Study population and design	1-2
Setting	5	Describe the setting, locations, and relevant dates, including periods of recruitment, exposure, follow-up, and data collection	Methods - Study population and design	1-2
Participants	6	(*a*) *Cohort study*—Give the eligibility criteria, and the sources and methods of selection of participants. Describe methods of follow-up<br>*Case-control study*—Give the eligibility criteria, and the sources and methods of case ascertainment and control selection. Give the rationale for the choice of cases and controls<br>*Cross-sectional study*—Give the eligibility criteria, and the sources and methods of selection of participants	Methods - Study population and design	2
		(*b*) *Cohort study*—For matched studies, give matching criteria and number of exposed and unexposed<br>*Case-control study*—For matched studies, give matching criteria and the number of controls per case	Methods - Study population and design	2
Variables	7	Clearly define all outcomes, exposures, predictors, potential confounders, and effect modifiers. Give diagnostic criteria, if applicable	Methods - Study population and design; Biomarker assessment; Covariates	Methods section paragraphs 2-6
Data sources/ measurement	8*	For each variable of interest, give sources of data and details of methods of assessment (measurement). Describe	Methods - Study	Methods section

		comparability of assessment methods if there is more than one group	population and design; Biomarker assessment; Covariates	paragraphs 2-6
Bias	9	Describe any efforts to address potential sources of bias	NA	
Study size	10	Explain how the study size was arrived at	Methods - Biomarker assessment	1
Quantitative variables	11	Explain how quantitative variables were handled in the analyses. If applicable, describe which groupings were chosen and why	Methods – Covariates; Statistical methods	Covariates – single paragraph; Statistical methods paragraph 3
Statistical methods	12	(*a*) Describe all statistical methods, including those used to control for confounding	Methods – Covariates; Statistical methods	Both subsections
		(*b*) Describe any methods used to examine subgroups and interactions	Methods – Statistical methods	Paragraphs 3-4
		(*c*) Explain how missing data were addressed	NA	
		(*d*) *Cohort study*—If applicable, explain how loss to follow-up was addressed<br>*Case-control study*—If applicable, explain how matching of cases and controls was addressed<br>*Cross-sectional study*—If applicable, describe analytical methods taking account of sampling strategy	Methods – Statistical methods	Paragraphs 1 and 3
		(*e*) Describe any sensitivity analyses	Methods – Statistical methods	4

Continued on next page

**Results**				
Participants	13*	(a) Report numbers of individuals at each stage of study—eg numbers potentially eligible, examined for eligibility, confirmed eligible, included in the study, completing follow-up, and analysed	Methods - Biomarker assessment	1
		(b) Give reasons for non-participation at each stage	Methods - Biomarker assessment	1
		(c) Consider use of a flow diagram	Supplement	S1 Figure
Descriptive data	14*	(a) Give characteristics of study participants (eg demographic, clinical, social) and information on exposures and potential confounders	Results	1
		(b) Indicate number of participants with missing data for each variable of interest	NA	
		(c) *Cohort study*—Summarise follow-up time (eg, average and total amount)	NA	
Outcome data	15*	*Cohort study*—Report numbers of outcome events or summary measures over time	NA	
		Case-control study—Report numbers in each exposure category, or summary measures of exposure	Table 2	Table 2
		Cross-sectional study—Report numbers of outcome events or summary measures	NA	
Main results	16	(*a*) Give unadjusted estimates and, if applicable, confounder-adjusted estimates and their precision (eg, 95% confidence interval). Make clear which confounders were adjusted for and why they were included	Figure 1; S6 Figure	NA
		(*b*) Report category boundaries when continuous variables were categorized	Table 1	NA
		(*c*) If relevant, consider translating estimates of relative risk into absolute risk for a meaningful time period	NA	
Other analyses	17	Report other analyses done—eg analyses of subgroups and interactions, and sensitivity analyses	Results - Plasma phospholipid PUFAs in early to mid-pregnancy and subsequent GDM risk	1
**Discussion**				
Key results	18	Summarise key results with reference to study objectives	Discussion	1
Limitations	19	Discuss limitations of the study, taking into account sources of potential bias or imprecision. Discuss both direction and magnitude of any potential bias	Discussion – Strengths and limitations	2
Interpretation	20	Give a cautious overall interpretation of results considering	Discussion –	NA

		objectives, limitations, multiplicity of analyses, results from similar studies, and other relevant evidence	subsections on Comparison with other studies on GDM; Comparison with other studies on T2DM; Biological plausibility and implications	
Generalisability	21	Discuss the generalisability (external validity) of the study results	Discussion – Strengths and limitations	2
**Other information**				
Funding	22	Give the source of funding and the role of the funders for the present study and, if applicable, for the original study on which the present article is based	Title page - funding	NA

*Give information separately for cases and controls in case-control studies and, if applicable, for exposed and unexposed groups in cohort and cross-sectional studies.

Note: An Explanation and Elaboration article discusses each checklist item and gives methodological background and published examples of transparent reporting. The STROBE checklist is best used in conjunction with this article (freely available on the Web sites of PLoS Medicine at http://www.plosmedicine.org/, Annals of Internal Medicine at http://www.annals.org/, and Epidemiology at http://www.epidem.com/). Information on the STROBE Initiative is available at www.strobe-statement.org.
