# Supplementary material for: Plasma phospholipid n-3 and n-6 polyunsaturated fatty acids in relation to cardiometabolic markers and gestational diabetes: A longitudinal study within the prospective NICHD Fetal Growth Studies
Source: PLoS Med. 2019 Sep 13;16(9):e1002910. doi: 10.1371/journal.pmed.1002910 (PMC6743768; doi:10.1371/journal.pmed.1002910)
Supplement: S1 Protocol — (PDF) [file pmed.1002910.s002.pdf]

S1 Protocol

Plasma phospholipid n-3 and n-6 polyunsaturated fatty acids in relation to cardiometabolic markers and gestational diabetes: A longitudinal study within the prospective NICHD Fetal Growth Studies

Proposer: Yeyi Zhu,[1,2] Cuilin Zhang[3]

Writing team members: Mengying Li,[3] Mohammad L. Rahman,[4] Stefanie N. Hinkle,[3] Jing Wu,[5] Yuan Lin,[6] Huixia Yang,[7] Michael Y. Tsai,[8] Assiamira Ferrara[1]

[1]Division of Research, Kaiser Permanente Northern California, Oakland, CA
[2]Department of Epidemiology & Biostatistics, University of California, San Francisco, CA
[3]Division of Intramural Population Health Research, *Eunice Kennedy Shriver* National Institute of Child Health and Human Development, Bethesda, MD
[4]Harvard Medical School, Department of Population Medicine and Harvard Pilgrim Health Care Institute
[5]Glotech Inc., Bethesda, MD
[6]Department of Epidemiology, Richard M. Fairbanks School of Public Health, Indiana University, Indianapolis, Indiana, USA
[7]Department of Obstetrics and Gynecology, Peking University First Hospital, Beijing, China
[8]Department of Laboratory Medicine and Pathology, University of Minnesota, Minneapolis, MN

Background

Gestational diabetes (GDM) has emerged as the most common metabolic complication affecting 7-25% of pregnancies worldwide.[1,2] Emerging experimental data have linked altered fatty acids composition to exacerbation of insulin resistance and β-cell dysfunction,[3-5] suggesting additional pathways underlying the etiology of hyperglycemia. However, epidemiological data linking dietary intakes of polyunsaturated fatty acids (PUFAs) to glucose homeostasis and diabetes risk remain equivocal. Notably, dietary assessment is subject to inherent recall bias and measurement errors and levels of several circulating long-chain PUFA derivatives are functions of both exogenous (via dietary intake) and endogenous (via *de novo* lipogenesis) origins, calling for objective assessment of individual plasma phospholipid PUFAs. Furthermore, pregnancy is a unique window of drastic physiological changes; however, data on plasma phospholipid PUFAs during early to mid-pregnancy in relation to subsequent risk of GDM is lacking.

Objectives

1. Examine the prospective associations between plasma phospholipid PUFAs in early to mid-pregnancy and subsequent risk of GDM at gestational weeks 24-28.
2. Examine the prospective associations between plasma phospholipid PUFAs at gestational weeks 10-14 and a panel of glucose metabolism and cardiovascular markers at gestational weeks 15-26.
3. Characterize the longitudinal profiles of plasma phospholipid PUFAs across gestation**.**

Methods

Study sample

This study will utilize data from the prospective NICHD Fetal Growth Study-Singletons Cohort (n = 2,802). A total of 107 GDM cases were identified via medical record review. A random sample of non-GDM controls were matched to cases according to age (± 2 years), race/ethnicity (non-Hispanic White, non-Hispanic Black, Hispanic, Asian/Pacific Islander), and gestational age at blood sample collection (± 2 weeks). Non-GDM controls were matched 2:1 to GDM cases for blood specimens collected at enrollment (10-14 weeks) and study visit 1 (15-26 weeks), and 1:1 at study visits 2 (24-29) and 4 (34-37).

Exposure: Plasma phospholipid PUFAs

1. Individual PUFAs: Plasma phospholipid PUFAs measured using a Hewlett Packard 5890 gas chromatography system included four n-3 PUFAs: 18:3n-3 (ALA), 20:5n-3 (eicosapentaenoic acid, EPA), 22:5n-3 (docosapentaenoic acid, n-3 DPA), and 22:6n-3 (docosahexaenoic acid, DHA); and seven n-6 PUFAs: 18:2n-6 (LA), 18:3n-6 (gamma-linoleic acid, GLA), 20:2n-6 (eicosadienoic acid, EDA), 20:3n-6 (dihomo-gamma-linolenic acid, DGLA), 20:4n-6 (arachidonic acid, AA), 22:4n-6 (docosatetraenoic acid, DTA), and 22:5n-6 (docosapentaenoic acid, n-6 DPA). The content of individual plasma phospholipid PUFA was expressed as a percentage (%) of the total phospholipid fatty acids.
2. PUFA subclasses: n-3 PUFAs and n-6 PUFAs
3. PUFA ratios as indicators of fatty acid elongase and desaturase enzyme activities: 18:3n-6/18:2n-6 (GLA/LA) indicating Δ6-desaturase activity catalyzing the conversion of LA to GLA, 20:4n-6/20:3n-6 (AA/DGLA) indicating Δ5-desaturase activity catalyzing the conversion of DGLA to AA, 20:3n-6/18:2n-6 (DGLA/LA) indicating the conversion of LA to DGLA.
4. Measurement time points: gestational weeks 10-14, 15-26, 23-31, and 33-39 weeks (without overlapping within individual subject). We will focus on the first two time points which were prior to diagnosis of GDM.

Outcome: 107 GDM cases were ascertained via medical record review according to the Carpenter and Coustan criteria at gestational weeks 24-28. 214 non-GDM randomly selected controls were matched 2:1 to cases on age (± 2 years), race/ethnicity (non-Hispanic White, non-Hispanic Black, Hispanic, Asian/Pacific Islander), and gestational week of blood sample collection (± 2 weeks).

Covariates: Potential covariates include maternal demographic, lifestyle, and clinical factors: maternal education, insurance, parity, family history of diabetes, smoking and alcohol consumption before pregnancy, and pre-pregnancy BMI categories. Selection of covariates will be both knowledge-based and statistically based (covariates meeting the inclusion criteria of ≥10% change in the main effect estimates will be retained in the final multivariable model). We will also include the two matching factors: maternal age (years) and gestational week of blood collection (weeks), which were matched between cases and controls within a certain range.

Statistical analysis:

1. Compare participant characteristics and plasma phospholipid PUFA concentrations at enrollment (10-14 weeks) and study visit 1 (15-26 weeks) between GDM cases and non-GDM controls using
   - linear mixed models with associated likelihood ratio tests for continuous variables
   - logistic regression with generalized estimating equations for categorical variables.

2. Characterize and plot the distribution of plasma phospholipid PUFA concentrations and PUFA ratios across gestational intervals of 3-4 weeks across gestation by GDM/control status.

3. Correlations of individual and subclasses of plasma phospholipid PUFAs and PUFA ratios at gestational weeks 10-14 with markers of glucose homeostasis (fasting plasma glucose, insulin, C-peptide, hs-CRP, and HOMA-IR) and cardiometabolic risk (adiponectin, leptin, total cholesterol, HDL-C, LDL-C, and triglycerides) at the subsequent visit (gestational weeks 15-26).

4. Construct multivariable conditional logistic regression models to assess the
   - associations of individual circulating PUFAs and FA ratios at the first two visits (gestational weeks 10-14 and 15-26) with risk of GDM, respectively, adjusting for afore-mentioned covariates. Parameter each PUFA and FA ratio as a categorical variable in quartiles based on the distribution among controls and also a continuous variable per standard deviation.
   - risk estimates associated with joint categories of high or low n-3 and n-6 PUFAs determined as above or below the respective median at the first two visits including an interaction term for continuous n-3 and n-6 PUFAs.
   - All models adjusted for covariates: age (years), gestational age at blood collection (weeks), parity (nulliparous, multiparous), family history of diabetes (yes, no), and pre-pregnancy body mass index (<25.0, 25.0-29.9, 30.0-34.9, 35.0-44.9 kg/m^2).

5. Sensitivity analysis to examine the potential effect modification by pre-pregnancy obesity status, family history of diabetes, and race/ethnicity.

References

1. Guariguata L, Linnenkamp U, Beagley J, Whiting DR, Cho NH. Global estimates of the prevalence of hyperglycaemia in pregnancy. *Diabetes research and clinical practice.* 2014;103(2):176-185.
2. Ferrara A. Increasing prevalence of gestational diabetes mellitus: a public health perspective. *Diabetes Care.* 2007;30 Suppl 2:S141-146.
3. Boden G, She P, Mozzoli M, et al. Free fatty acids produce insulin resistance and activate the proinflammatory nuclear factor-κB pathway in rat liver. *Diabetes.* 2005;54(12):3458-3465.
4. Koves TR, Ussher JR, Noland RC, et al. Mitochondrial overload and incomplete fatty acid oxidation contribute to skeletal muscle insulin resistance. *Cell metabolism.* 2008;7(1):45-56.
5. Storlien LH, Jenkins AB, Chisholm DJ, Pascoe WS, Khouri S, Kraegen EW. Influence of dietary fat composition on development of insulin resistance in rats: relationship to muscle triglyceride and ω-3 fatty acids in muscle phospholipid. *Diabetes.* 1991;40(2):280-289.
