# Supplementary material for: Plasma phospholipid n-3 and n-6 polyunsaturated fatty acids in relation to cardiometabolic markers and gestational diabetes: A longitudinal study within the prospective NICHD Fetal Growth Studies
Source: PLoS Med. 2019 Sep 13;16(9):e1002910. doi: 10.1371/journal.pmed.1002910 (PMC6743768; doi:10.1371/journal.pmed.1002910)

S1 Fig. Flow chart of the nested case-control study, within the NICHD Fetal Growth Studies-Singleton Cohort

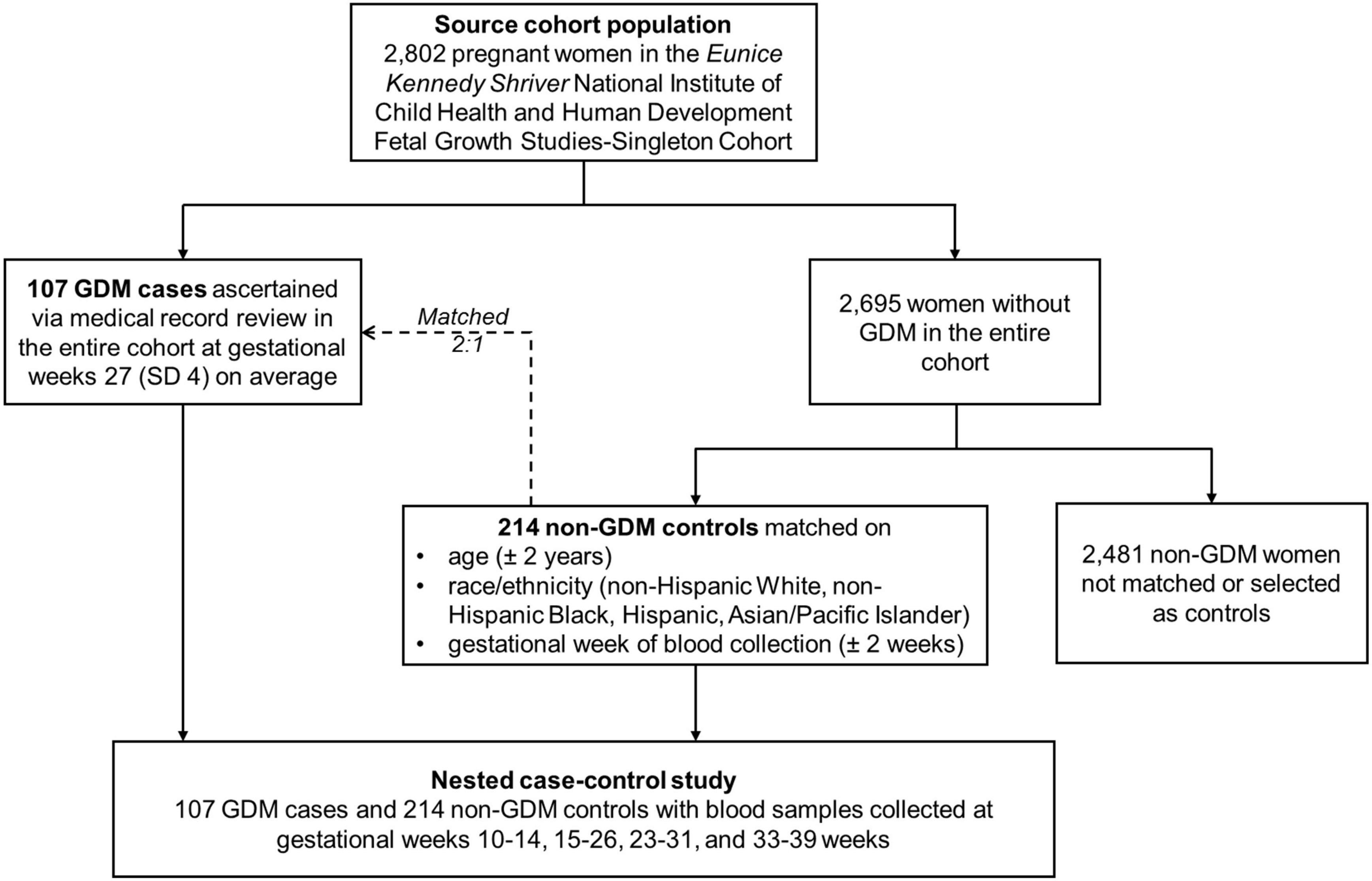

Supplement: S1 Fig — NICHD, National Institute of Child Health and Human Development. (PDF) [file pmed.1002910.s003.pdf]
