# Supplementary material for: Plasma phospholipid n-3 and n-6 polyunsaturated fatty acids in relation to cardiometabolic markers and gestational diabetes: A longitudinal study within the prospective NICHD Fetal Growth Studies
Source: PLoS Med. 2019 Sep 13;16(9):e1002910. doi: 10.1371/journal.pmed.1002910 (PMC6743768; doi:10.1371/journal.pmed.1002910)

S2 Fig. Metabolic pathways and major exogenous (dietary) and endogenous (lipogenesis) sources of polyunsaturated fatty acids.

PUFA, polyunsaturated fatty acids. Fatty acid elongase and desaturase are denoted in grey squares. Sources of PUFAs are in italic.

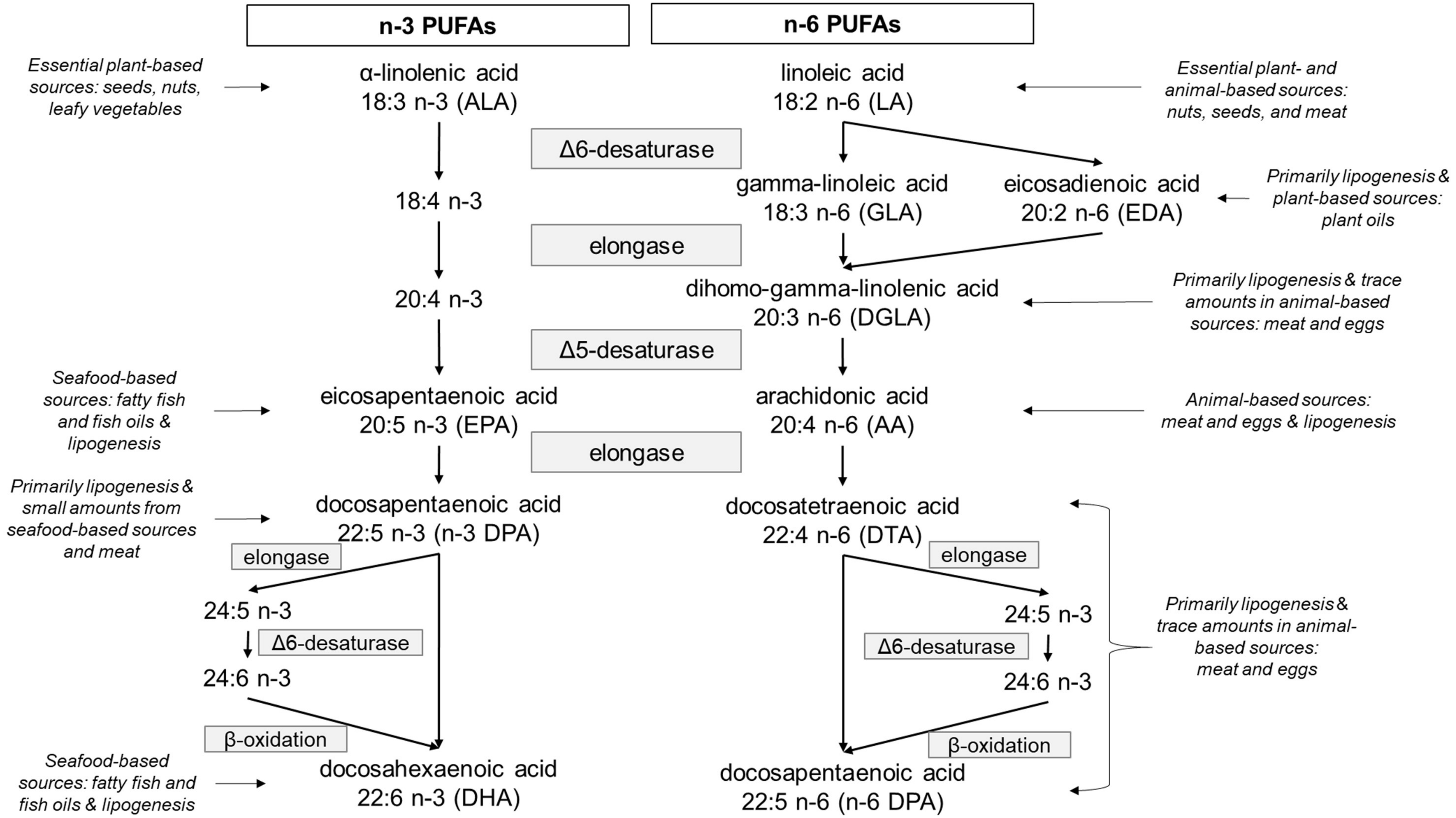

Supplement: S2 Fig — (PDF) [file pmed.1002910.s004.pdf]
