# Supplementary material for: Plasma phospholipid n-3 and n-6 polyunsaturated fatty acids in relation to cardiometabolic markers and gestational diabetes: A longitudinal study within the prospective NICHD Fetal Growth Studies
Source: PLoS Med. 2019 Sep 13;16(9):e1002910. doi: 10.1371/journal.pmed.1002910 (PMC6743768; doi:10.1371/journal.pmed.1002910)

S3 Fig. Longitudinal profiles (mean ± standard errors, %) of plasma phospholipid n-3 PUFA and n-6 PUFA throughout pregnancy according to gestational-age intervals among women with and without GDM

PUFA, polyunsaturated fatty acid.

*P <0.05 for case-control comparisons obtained by linear mixed models with associated likelihood ratio tests accounting for matched case-control pairs at each gestational-age interval.

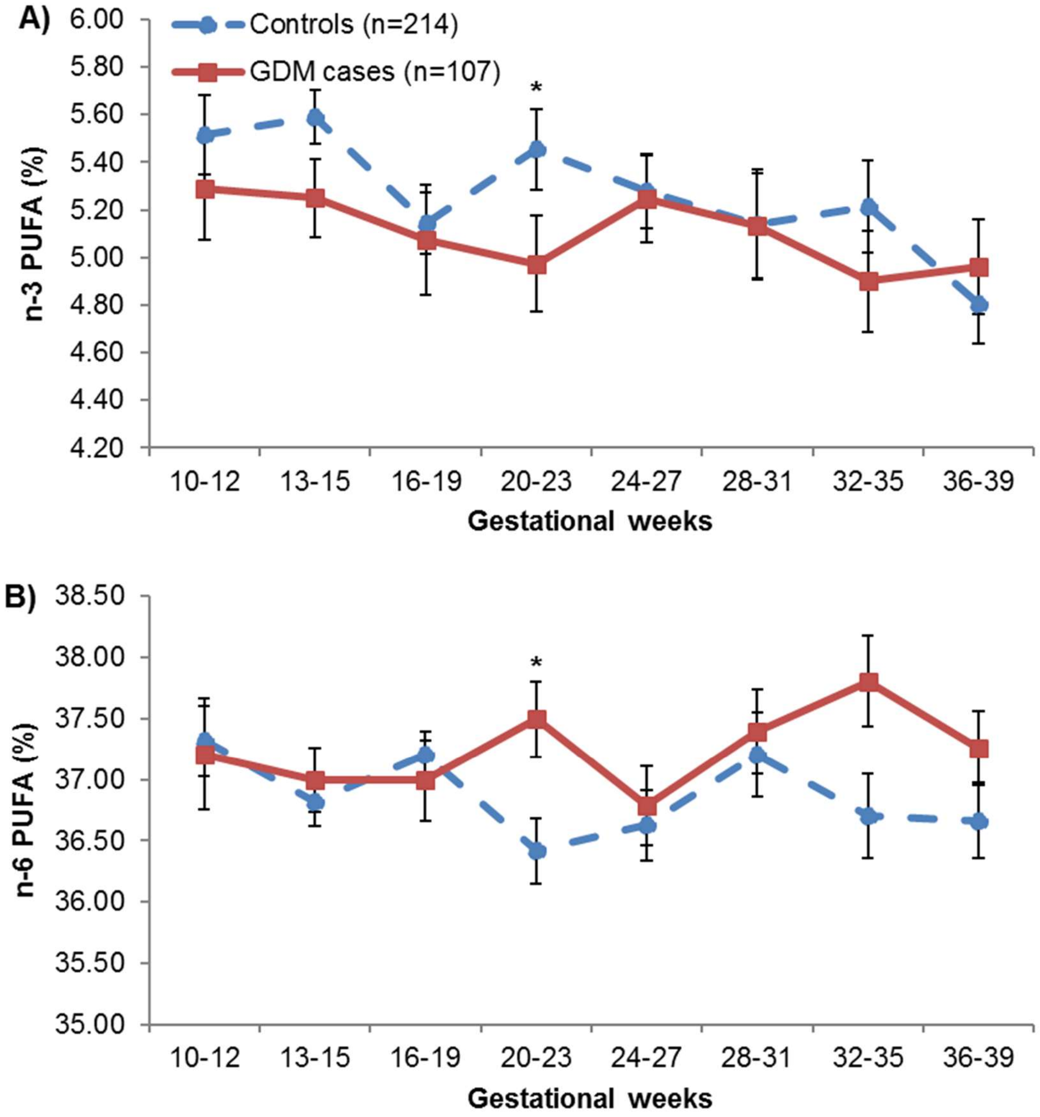

Supplement: S3 Fig — GDM, gestational diabetes mellitus; PUFA, polyunsaturated fatty acid. (PDF) [file pmed.1002910.s005.pdf]
