# Supplementary material for: Plasma phospholipid n-3 and n-6 polyunsaturated fatty acids in relation to cardiometabolic markers and gestational diabetes: A longitudinal study within the prospective NICHD Fetal Growth Studies
Source: PLoS Med. 2019 Sep 13;16(9):e1002910. doi: 10.1371/journal.pmed.1002910 (PMC6743768; doi:10.1371/journal.pmed.1002910)
Supplement: S4 Fig — GDM, gestational diabetes mellitus; PUFA, polyunsaturated fatty acid. (PDF) [file pmed.1002910.s006.pdf]

S4 Fig. Longitudinal profiles (mean ± standard errors, %) of plasma phospholipid PUFA ratios throughout pregnancy according to gestational-age intervals among women with and without GDM.

DGLA, dihomo-gamma-linolenic acid; LA, linoleic acid; PUFA, polyunsaturated fatty acid.

$^{*}P$ <0.05; $^{**}P$ <0.01; $^{***}P$ <0.001 for case-control comparisons obtained by linear mixed models with associated likelihood ratio tests accounting for matched case-control pairs at each gestational-age interval.

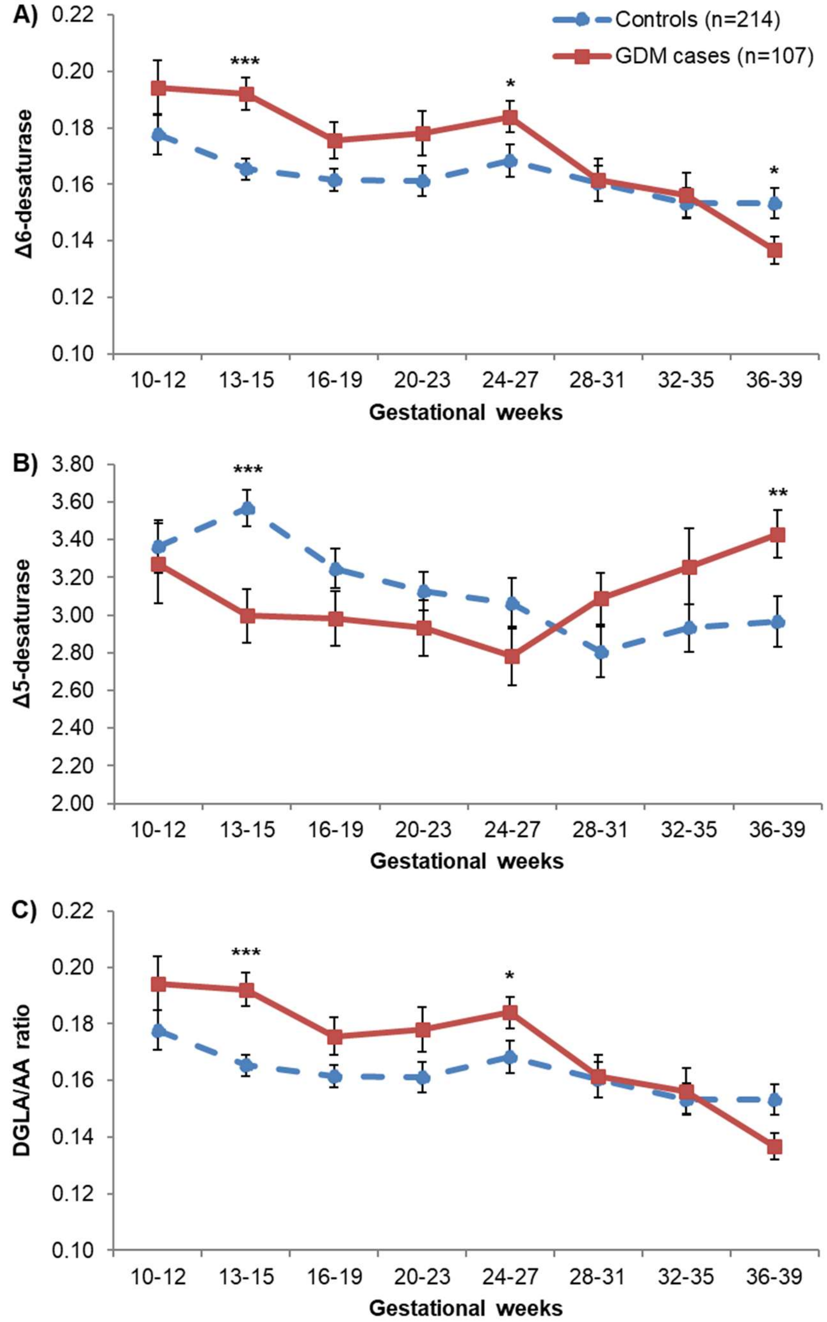

Number of specimens

Controls	62	155	119	88	67	43	45	58
Cases	32	76	55	43	56	45	38	51
