# Supplementary material for: Plasma phospholipid n-3 and n-6 polyunsaturated fatty acids in relation to cardiometabolic markers and gestational diabetes: A longitudinal study within the prospective NICHD Fetal Growth Studies
Source: PLoS Med. 2019 Sep 13;16(9):e1002910. doi: 10.1371/journal.pmed.1002910 (PMC6743768; doi:10.1371/journal.pmed.1002910)

S5 Fig. Heat map of the correlation matrix of plasma phospholipid n-3 PUFA, n-6 PUFA, and PUFA ratios with glucose metabolism and cardiometabolic markers at gestational weeks 10-14 among non-GDM controls.

The correlations were obtained by deriving Spearman correlation coefficients. Red represents a positive correlation and blue represents a negative correlation. AA, arachidonic acid; ALA, alpha-linolenic acid; DGLA, dihomo-gamma-linolenic acid; DHA, docosahexaenoic acid; DPA, docosapentaenoic acid; DTA, docosatetraenoic acid; EDA, eicosadienoic acid; EPA, eicosapentaenoic acid; GLA, gamma-linolenic acid; HDLD, high-density lipoprotein cholesterol; HMW-adiponectin, high molecular weight adiponectin; HOMA-IR, homeostasis model assessment of insulin resistance; hs-CRP, high sensitivity C-reactive protein; LA, linoleic acid; LDLD, low-density lipoprotein cholesterol; n-6 DPA, n-6 docosapentaenoic acid; PUFA, polyunsaturated fatty acid; TG, triglycerides; delta5D, 20:4n-6/20:3n-6; delta6D, 18:3n-6/18:2n-6.

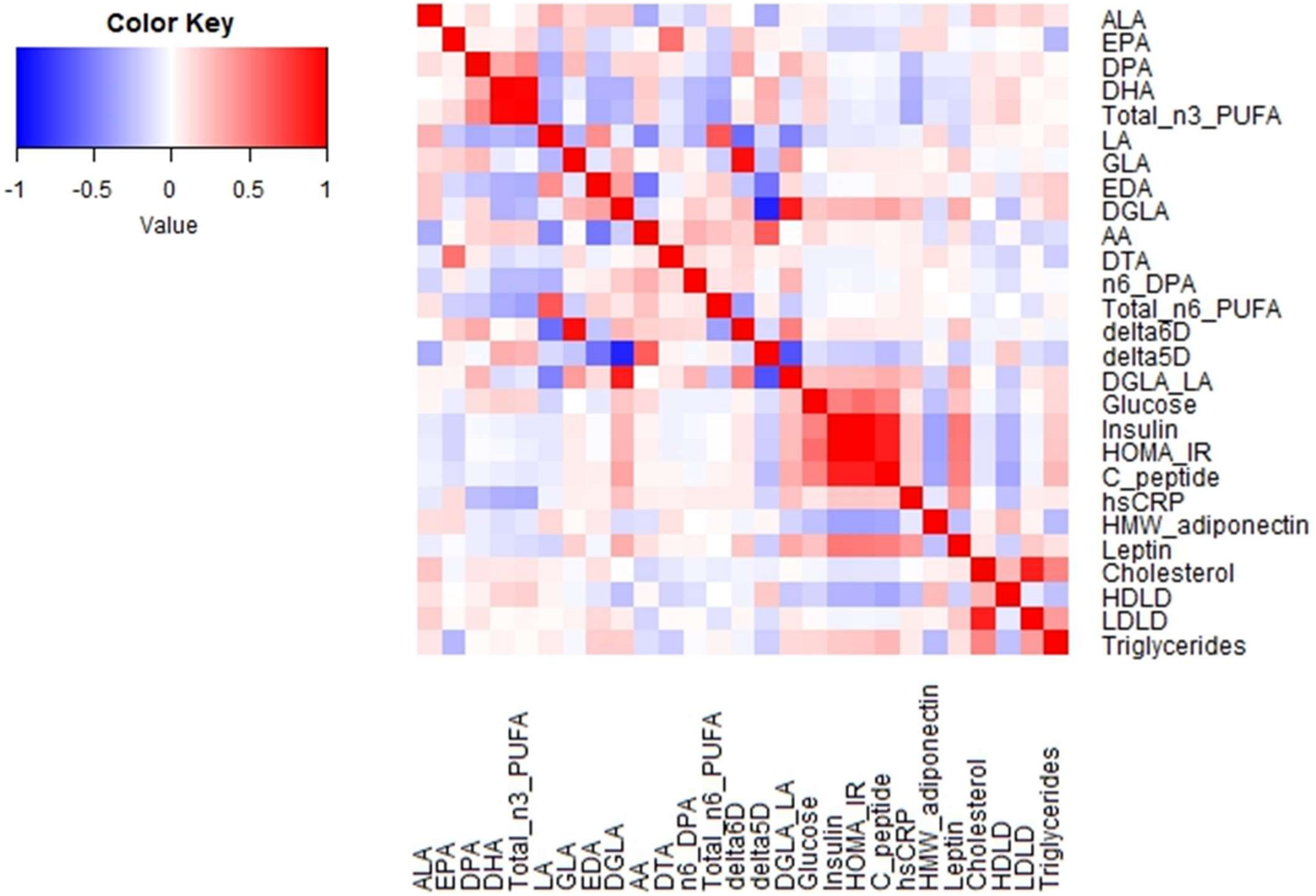

Supplement: S5 Fig — GDM, gestational diabetes mellitus; PUFA, polyunsaturated fatty acid. (PDF) [file pmed.1002910.s007.pdf]
