# Supplementary material for: Plasma phospholipid n-3 and n-6 polyunsaturated fatty acids in relation to cardiometabolic markers and gestational diabetes: A longitudinal study within the prospective NICHD Fetal Growth Studies
Source: PLoS Med. 2019 Sep 13;16(9):e1002910. doi: 10.1371/journal.pmed.1002910 (PMC6743768; doi:10.1371/journal.pmed.1002910)

S6 Fig. Unadjusted odds ratios (95% CIs) of GDM risk per one standard deviation increase in plasma phospholipid n-3 PUFA, n-6 PUFA, and PUFA ratios at gestational weeks 10-14 and 15-26

*, **, ***P value <0.05, 0.01, 0.001, after false discovery rate correction, respectively.

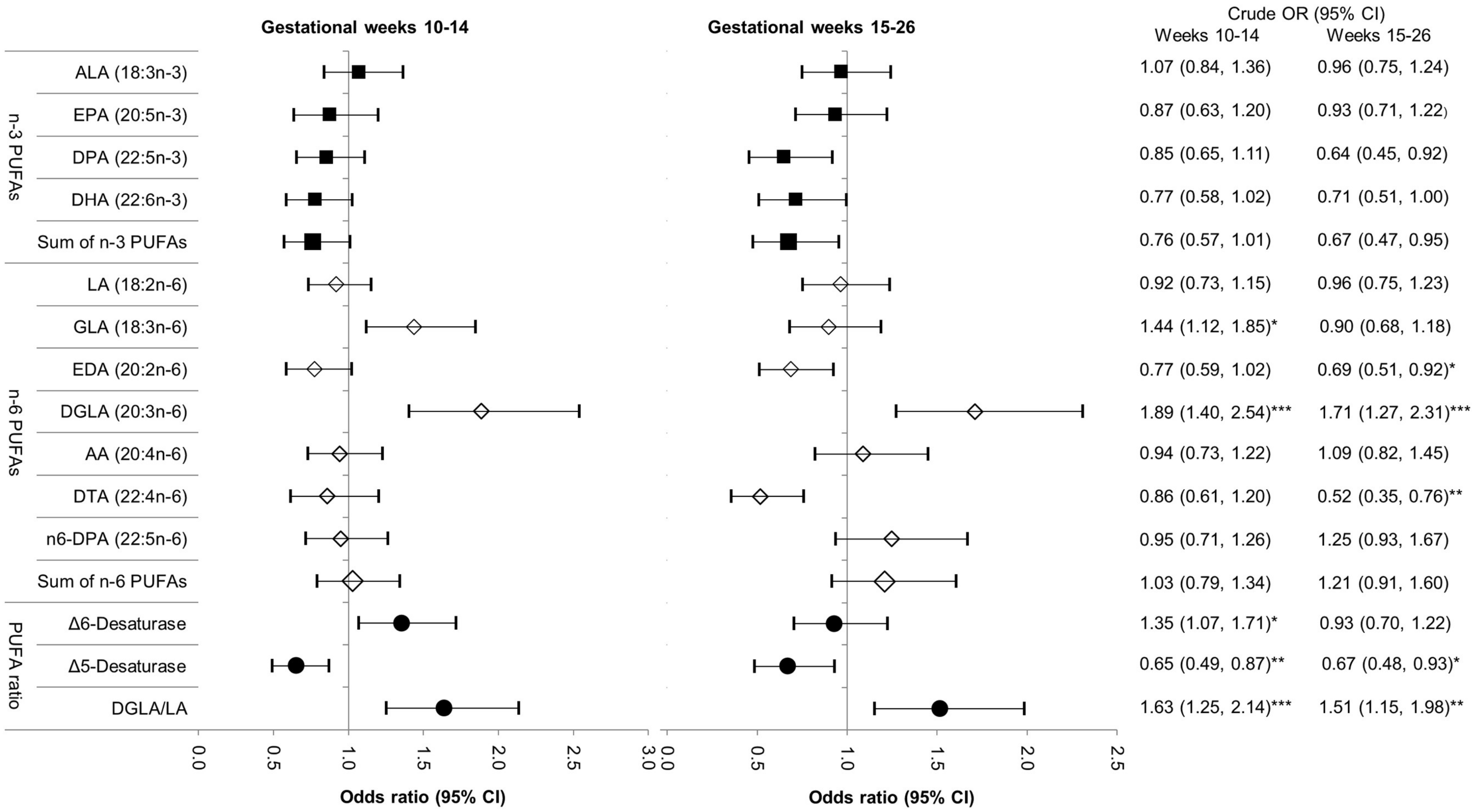

Supplement: S6 Fig — GDM, gestational diabetes mellitus; PUFA, polyunsaturated fatty acid. (PDF) [file pmed.1002910.s008.pdf]
