# Supplementary material for: Plasma phospholipid n-3 and n-6 polyunsaturated fatty acids in relation to cardiometabolic markers and gestational diabetes: A longitudinal study within the prospective NICHD Fetal Growth Studies
Source: PLoS Med. 2019 Sep 13;16(9):e1002910. doi: 10.1371/journal.pmed.1002910 (PMC6743768; doi:10.1371/journal.pmed.1002910)

S7 Fig. Adjusted odds ratios (95% CIs) of GDM risk in association with longitudinal changes of plasma phospholipid n-3 PUFA, n-6 PUFA, and PUFA ratios per one standard deviation increase from gestational weeks 10-14 to 15-26.

The risk estimates were adjusted for age (years), gestational age at blood collection (weeks), parity (nulliparous, multiparous), family history of diabetes (yes, no), and pre-pregnancy body mass index (<25.0, 25.0-29.9, 30.0-34.9, 35.0-44.9 kg/m^2).
$^{*, **, ***}$P value <0.05, 0.01, 0.001, after false discovery rate correction, respectively.

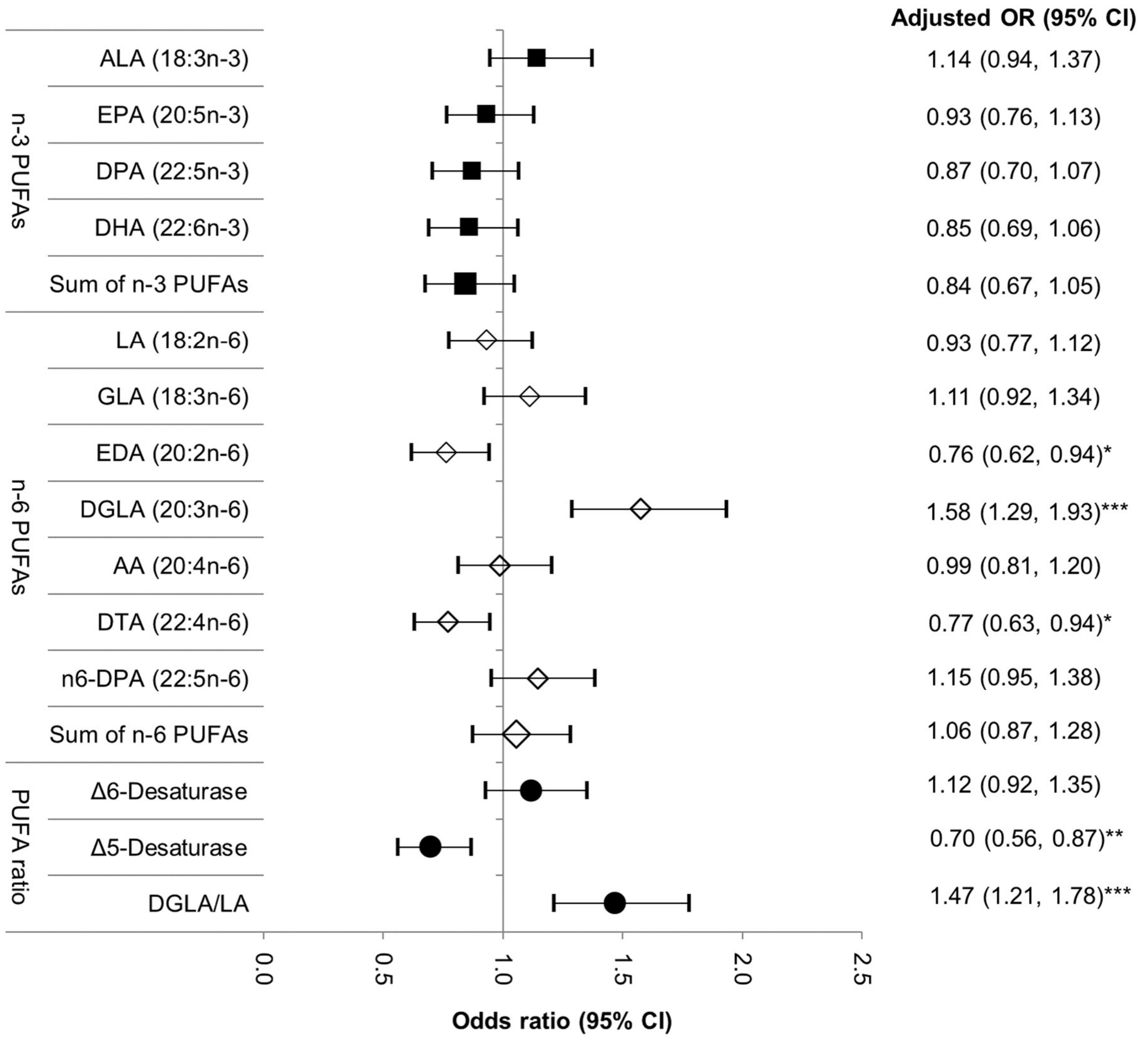

Supplement: S7 Fig — GDM, gestational diabetes mellitus; PUFA, polyunsaturated fatty acid. (PDF) [file pmed.1002910.s009.pdf]
