# Supplementary material for: Plasma phospholipid n-3 and n-6 polyunsaturated fatty acids in relation to cardiometabolic markers and gestational diabetes: A longitudinal study within the prospective NICHD Fetal Growth Studies
Source: PLoS Med. 2019 Sep 13;16(9):e1002910. doi: 10.1371/journal.pmed.1002910 (PMC6743768; doi:10.1371/journal.pmed.1002910)
Supplement: S1 Table — GDM, gestational diabetes mellitus; PUFA, polyunsaturated fatty acid. (PDF) [file pmed.1002910.s010.pdf]

S1 Table. Sensitivity analyses for the association of plasma phospholipid n-3 PUFA, n-6 PUFA, and PUFA ratios at gestational weeks 10-14 and 15-26 with GDM risk

	Gestational weeks 10-14		Gestational weeks 15-26	
	Model 2[1]	Model 3[2]	Model 2[1]	Model 3[2]
**n-3 PUFAs**				
18:3n-3 (alpha-linolenic acid, ALA)	1.24 (0.93, 1.65)	1.32 (0.98, 1.79)	1.10 (0.82, 1.46)	1.13 (0.84, 1.52)
20:5n-3 (eicosapentaenoic acid, EPA)	0.83 (0.58, 1.20)	0.76 (0.52, 1.12)	0.94 (0.72, 1.23)	0.97 (0.73, 1.28)
22:5n-3 (docosapentaenoic acid, DPA)	0.94 (0.69, 1.27)	0.77 (0.55, 1.08)	0.67 (0.45, 0.99)	0.68 (0.46, 1.01)
22:6n-3 (docosahexaenoic acid, DHA)	0.86 (0.62, 1.20)	0.82 (0.58, 1.16)	0.68 (0.45, 1.03)	0.68 (0.45, 1.05)
Total n-3 PUFAs	0.85 (0.61, 1.19)	0.78 (0.55, 1.12)	0.64 (0.42, 0.96)	0.65 (0.42, 0.99)
**n-6 PUFAs**				
18:2n-6 (linoleic acid, LA)	0.88 (0.68, 1.14)	1.24 (0.90, 1.72)	1.01 (0.75, 1.35)	1.08 (0.78, 1.48)
18:3n-6 (gamma-linoleic acid, GLA)	1.40 (1.05, 1.87)*	1.32 (1.01, 1.65)	0.92 (0.66, 1.29)	0.88 (0.63, 1.25)
20:2n-6 (eicosadienoic acid, EDA)	0.80 (0.59, 1.08)	0.94 (0.68, 1.30)	0.75 (0.55, 1.03)	0.76 (0.55, 1.05)
20:3n-6 (dihomo-gamma-linolenic acid, DGLA)	1.95 (1.37, 2.78)**	1.81 (1.25, 2.62)*	1.72 (1.22, 2.43)*	1.67 (1.17, 2.38)*
20:4n-6 (arachidonic acid, AA)	0.94 (0.71, 1.25)	0.90 (0.67, 1.22)	1.06 (0.77, 1.46)	1.07 (0.77, 1.48)
22:4n-6 (docosatetraenoic acid, DTA)	0.88 (0.61, 1.28)	0.82 (0.56, 1.20)	0.55 (0.37, 0.83)*	0.57 (0.38, 0.87)*
22:5n-6 (docosapentaenoic acid, n6-DPA)	0.98 (0.71, 1.36)	0.94 (0.66, 1.32)	1.31 (0.93, 1.85)	1.40 (1.04, 1.88)
Total n-6 PUFAs	0.95 (0.71, 1.29)	1.68 (1.09, 2.59)	1.30 (0.93, 1.83)	1.65 (1.08, 2.51)
**PUFA ratios**				
Δ6-Desaturase, 18:3n-6/18:2n-6	1.36 (1.03, 1.79)*	1.11 (0.82, 1.50)	0.95 (0.68, 1.32)	0.89 (0.63, 1.25)
Δ5-Desaturase, 20:4n-6/20:3n-6	0.65 (0.47, 0.90)*	0.67 (0.48, 0.94)	0.66 (0.45, 0.97)*	0.68 (0.46, 0.98)
DGLA/LA, 20:3n-6/18:2n-6	1.71 (1.26, 2.33)**	1.43 (1.01, 2.01)	1.51 (1.10, 2.06)*	1.46 (1.06, 2.02)

PUFA, polyunsaturated fatty acid. Values in the table are odds ratio (95% CI).

[1]The risk estimates were adjusted for age (years), gestational age at blood collection (weeks), parity (nulliparous, multiparous), family history of diabetes (yes, no), and pre-pregnancy body mass index (<25.0, 25.0-29.9, 30.0-34.9, 35.0-44.9 kg/m^2).

[2]Additionally adjusted for plasma phospholipid saturated fatty acids.

*, **P value <0.05, 0.01, after false discovery rate correction, respectively.
