# Supplementary material for: Plasma phospholipid n-3 and n-6 polyunsaturated fatty acids in relation to cardiometabolic markers and gestational diabetes: A longitudinal study within the prospective NICHD Fetal Growth Studies
Source: PLoS Med. 2019 Sep 13;16(9):e1002910. doi: 10.1371/journal.pmed.1002910 (PMC6743768; doi:10.1371/journal.pmed.1002910)
Supplement: S2 Table — GDM, gestational diabetes mellitus; PUFA, polyunsaturated fatty acid. (PDF) [file pmed.1002910.s011.pdf]

S2 Table. Odds ratios (95% CIs) for subsequent risk of GDM according to quartiles of plasma phospholipid n-3 PUFA, n-6 PUFA, and PUFA ratios at gestational weeks 10-14 and 15-26[1]

	Gestational weeks 10-14		Gestational weeks 15-26	
	Crude	Multivariable[1]	Crude	Multivariable[1]
**n-3 PUFAs**				
18:3n-3 (alpha-linolenic acid, ALA)				
Q1	Reference	Reference	Reference	Reference
Q2	1.32 (0.66, 2.63)	1.79 (0.79, 4.05)	0.59 (0.29, 1.20)	0.78 (0.34, 1.79)
Q3	1.38 (0.69, 2.78)	1.74 (0.79, 3.84)	1.23 (0.63, 2.44)	1.77 (0.79, 3.94)
Q4	1.30 (0.65, 2.60)	1.86 (0.83, 4.13)	0.76 (0.38, 1.55)	1.12 (0.48, 2.60)
P-for-trend[2]	0.609	0.493	0.700	0.542
20:5n-3 (eicosapentaenoic acid, EPA)				
Q1	Reference	Reference	Reference	Reference
Q2	0.47 (0.21, 1.06)	0.56 (0.24, 1.31)	0.57 (0.28, 1.16)	0.60 (0.27, 1.37)
Q3	0.55 (0.23, 1.34)	0.62 (0.24, 1.59)	0.78 (0.39, 1.56)	0.70 (0.30, 1.61)
Q4	0.82 (0.34, 1.99)	0.83 (0.32, 2.16)	0.42 (0.18, 0.98)	0.49 (0.19, 1.26)
P-for-trend[2]	0.998	0.852	0.117	0.275
22:5n-3 (docosapentaenoic acid, DPA)				
Q1	Reference	Reference	Reference	Reference
Q2	1.10 (0.55, 2.20)	0.92 (0.41, 2.03)	0.71 (0.34, 1.46)	0.72 (0.31, 1.68)
Q3	0.67 (0.31, 1.44)	0.53 (0.22, 1.28)	0.63 (0.30, 1.33)	0.79 (0.34, 1.82)
Q4	0.81 (0.38, 1.75)	1.13 (0.46, 2.79)	0.28 (0.11, 0.72)	0.29 (0.10, 0.85)
P-for-trend[2]	0.597	0.852	0.035	0.120
22:6n-3 (docosahexaenoic acid, DHA)				
Q1	Reference	Reference	Reference	Reference
Q2	0.66 (0.32, 1.35)	0.90 (0.42, 1.93)	0.82 (0.41, 1.63)	0.95 (0.43, 2.09)
Q3	0.79 (0.41, 1.54)	0.93 (0.44, 1.94)	0.46 (0.21, 1.03)	0.52 (0.20, 1.32)
Q4	0.43 (0.20, 0.96)	0.63 (0.25, 1.56)	0.61 (0.27, 1.39)	0.69 (0.25, 1.93)
P-for-trend[2]	0.230	0.672	0.170	0.406
Total n-3 PUFAs				
Q1	Reference	Reference	Reference	Reference
Q2	0.85 (0.43, 1.67)	1.03 (0.49, 2.17)	0.77 (0.39, 1.50)	0.83 (0.38, 1.79)
Q3	0.56 (0.26, 1.20)	0.77 (0.31, 1.87)	0.49 (0.21, 1.13)	0.55 (0.20, 1.55)
Q4	0.57 (0.28, 1.15)	0.59 (0.26, 1.32)	0.36 (0.15, 0.83)	0.36 (0.14, 0.98)
P-for-trend[2]	0.230	0.493	0.035	0.120
**n-6 PUFAs**				
18:2n-6 (linoleic acid, LA)				
Q1	Reference	Reference	Reference	Reference
Q2	0.80 (0.41, 1.56)	0.79 (0.36, 1.71)	0.82 (0.42, 1.60)	0.87 (0.41, 1.85)
Q3	1.01 (0.54, 1.90)	1.03 (0.50, 2.12)	0.78 (0.39, 1.55)	0.89 (0.40, 1.97)
Q4	0.68 (0.35, 1.31)	0.58 (0.27, 1.26)	0.91 (0.47, 1.79)	0.93 (0.41, 2.10)
P-for-trend[2]	0.483	0.321	0.936	0.884
18:3n-6 (gamma-linoleic acid, GLA)				
Q1	Reference	Reference	Reference	Reference
Q2	1.40 (0.65, 3.01)	1.23 (0.52, 2.89)	1.25 (0.61, 2.55)	0.98 (0.45, 2.18)
Q3	2.12 (1.03, 4.36)	1.36 (0.61, 3.06)	1.27 (0.64, 2.50)	1.03 (0.47, 2.27)
Q4	2.63 (1.26, 5.49)	2.53 (1.12, 5.68)	0.89 (0.41, 1.91)	0.90 (0.37, 2.18)
P-for-trend[2]	0.020	0.068	0.936	0.884
20:2n-6 (eicosadienoic acid, EDA)				
Q1	Reference	Reference	Reference	Reference

Q2	0.48 (0.24, 0.95)	0.61 (0.29, 1.28)	0.80 (0.40, 1.60)	0.91 (0.40, 2.06)
Q3	0.58 (0.29, 1.16)	0.65 (0.30, 1.38)	0.48 (0.23, 1.01)	0.58 (0.25, 1.32)
Q4	0.49 (0.23, 1.03)	0.53 (0.24, 1.18)	0.49 (0.23, 1.05)	0.62 (0.26, 1.49)
P-for-trend[2]	0.275	0.312	0.109	0.416
20:3n-6 (dihomo-gamma-linolenic acid, DGLA)				
Q1	Reference	Reference	Reference	Reference
Q2	1.79 (0.83, 3.89)	1.59 (0.67, 3.75)	1.73 (0.64, 4.63)	1.67 (0.56, 4.92)
Q3	2.03 (0.89, 4.59)	2.36 (0.97, 5.75)	4.27 (1.64, 11.2)	3.76 (1.28, 11.1)
Q4	3.98 (1.77, 8.93)	3.42 (1.37, 8.54)	5.53 (2.07, 14.8)	5.12 (1.70, 15.4)
P-for-trend[2]	0.008	0.056	0.001	0.008
20:4n-6 (arachidonic acid, AA)				
Q1	Reference	Reference	Reference	Reference
Q2	0.74 (0.40, 1.38)	0.61 (0.30, 1.25)	0.71 (0.35, 1.43)	0.72 (0.31, 1.66)
Q3	0.52 (0.25, 1.09)	0.43 (0.18, 1.04)	0.76 (0.35, 1.62)	0.83 (0.34, 2.03)
Q4	0.70 (0.36, 1.39)	0.63 (0.30, 1.32)	1.06 (0.51, 2.23)	1.04 (0.45, 2.43)
P-for-trend[2]	0.325	0.312	0.939	0.884
22:4n-6 (docosatetraenoic acid, DTA)				
Q1	Reference	Reference	Reference	Reference
Q2	0.57 (0.26, 1.24)	0.72 (0.31, 1.68)	0.56 (0.29, 1.08)	0.76 (0.35, 1.65)
Q3	0.43 (0.18, 1.02)	0.44 (0.16, 1.20)	0.46 (0.21, 0.98)	0.47 (0.20, 1.13)
Q4	0.56 (0.22, 1.40)	0.56 (0.20, 1.59)	0.22 (0.09, 0.56)	0.30 (0.11, 0.83)
P-for-trend[2]	0.294	0.321	0.004	0.060
22:5n-6 (docosapentaenoic acid, n6-DPA)				
Q1	Reference	Reference	Reference	Reference
Q2	2.29 (1.11, 4.72)	2.41 (1.04, 5.58)	1.23 (0.57, 2.65)	1.40 (0.57, 3.44)
Q3	1.53 (0.73, 3.24)	2.11 (0.89, 5.03)	1.41 (0.66, 2.99)	2.16 (0.87, 5.33)
Q4	1.46 (0.62, 3.41)	1.61 (0.62, 4.14)	1.77 (0.78, 4.00)	2.39 (0.93, 6.15)
P-for-trend[2]	0.483	0.321	0.322	0.128
Total n-6 PUFAs				
Q1	Reference	Reference	Reference	Reference
Q2	0.77 (0.39, 1.52)	0.51 (0.23, 1.1 = 0.603)	1.25 (0.58, 2.68)	1.32 (0.55, 3.19)
Q3	0.97 (0.47, 2.02)	0.77 (0.32, 1.85)	0.97 (0.45, 2.09)	0.91 (0.38, 2.19)
Q4	0.91 (0.44, 1.86)	0.67 (0.29, 1.55)	1.48 (0.67, 3.26)	1.79 (0.69, 4.61)
P-for-trend[2]	0.894	0.460	0.757	0.624
**PUFA ratios**				
Δ6-Desaturase, 18:3n-6/18:2n-6				
Q1	Reference	Reference	Reference	Reference
Q2	1.21 (0.58, 2.54)	0.88 (0.38, 2.02)	2.06 (1.00, 4.23)	2.32 (0.97, 5.52)
Q3	1.49 (0.73, 3.07)	0.93 (0.42, 2.09)	1.65 (0.79, 3.43)	1.49 (0.64, 3.49)
Q4	2.03 (1.01, 4.08)	1.86 (0.86, 4.03)	0.95 (0.42, 2.18)	1.01 (0.39, 2.60)
P-for-trend[2]	0.047	0.114	0.810	0.775
Δ5-Desaturase, 20:4n-6/20:3n-6				
Q1	Reference	Reference	Reference	Reference
Q2	0.86 (0.47, 1.57)	1.08 (0.53, 2.18)	0.62 (0.30, 1.25)	0.69 (0.31, 1.55)
Q3	0.41 (0.20, 0.87)	0.45 (0.19, 1.05)	0.83 (0.42, 1.64)	0.92 (0.42, 1.98)
Q4	0.37 (0.17, 0.81)	0.35 (0.14, 0.84)	0.27 (0.11, 0.68)	0.22 (0.07, 0.67)
P-for-trend[2]	0.015	0.030	0.048	0.078
DGLA/LA, 20:3n-6/18:2n-6				

Q1	Reference	Reference	Reference	Reference
Q2	3.18 (1.36, 7.45)	3.67 (1.45, 9.28)	1.93 (0.73, 5.13)	1.83 (0.60, 5.53)
Q3	2.17 (0.92, 5.12)	2.10 (0.83, 5.36)	3.01 (1.16, 7.83)	2.24 (0.72, 6.97)
Q4	5.80 (2.40, 14.0)	5.89 (2.25, 15.4)	4.72 (1.85, 12.1)	4.35 (1.47, 12.8)
P-for-trend[2]	0.076	0.221	0.726	0.752

PUFA, polyunsaturated fatty acid; Q, quartile.
[1]Adjusted for age (years), gestational age at blood collection (weeks), parity (nulliparous, multiparous), family history of diabetes (yes, no), and pre-pregnancy body mass index (<25.0, 25.0-29.9, 30.0-34.9, 35.0-44.9 kg/m^2.
[2]P-for-trend was corrected using the false discovery rate method.
